# Brain metabolic and network profiles characterized by comorbid depressive symptoms and sleep disturbances in Parkinson’s disease: an ^18^F-FDG PET study

**DOI:** 10.3389/fnagi.2026.1901625

**Published:** 2026-09-09

**Authors:** Kaidi Li, Caihua Bao, Yinxia Wang, Chunyu Zhang, Xin’ai Wu, Yaping Du, Yikai Shu, Yadong Fan, Pusheng Quan, Zhijun Li, Juan He

**Affiliations:** 1 Department of Neurology, Affiliated Hospital of Inner Mongolia Medical University, Hohhot, Inner Mongolia, China; 2 School of Traditional Chinese Medicine, Beijing University of Chinese Medicine, Beijing, China; 3 Department of Neurology, Tongliao City People’s Hospital, Tongliao, Inner Mongolia, China; 4 Department of Nuclear Medicine, Affiliated Hospital of Inner Mongolia Medical University, Hohhot, Inner Mongolia, China; 5 The First Affiliated Hospital, and College of Clinical Medicine of Henan University of Science and Technology, Luoyang, Henan, China; 6 The First Affiliated Hospital of Hebei North University, Zhangjiakou, Hebei, China; 7 Human Anatomy Teaching and Research Section, School of Basic Medicine, Inner Mongolia Medical University, Hohhot, Inner Mongolia, China

**Keywords:** 18F-FDG PET, brain metabolism, depressive symptoms, Parkinson’s disease, sleep disturbances, brain network

## Abstract

**Background:**

Parkinson’s disease (PD) is a globally increasing neurodegenerative disorder. Its complex, multisystem pathophysiology extends beyond dopaminergic neuron loss and gives rise to diverse clinical manifestations. This exploratory study examined brain metabolic patterns in patients with PD and depressive symptoms (PD-Dep) or sleep disturbances (PD-SD).

**Methods:**

Twenty-eight patients with PD assessed between December 2019 and January 2024 were included. Sixteen met the HAMD threshold for PD-Dep, 21 met the PSQI threshold for PD-SD, and nine met both criteria; the cohorts were therefore overlapping symptom-defined groups. Seventeen healthy participants served as controls (HC). All participants underwent 18F-FDG PET. Voxel-wise group contrasts and exploratory AAL-based partial correlations with HAMD, PSQI, and UPDRS-III were examined.

**Results:**

At the prespecified liberal voxel-wise cluster-forming threshold, both symptom-defined PD groups showed extensive frontoparietal SUVR reductions relative to HC; these spatial findings should be considered exploratory. In PD-Dep, nominal HAMD associations included cortical and subcortical regions, whereas UPDRS-III associations were concentrated in frontoparietal regions. In PD-SD, nominal PSQI associations showed negative frontal/somatomotor and positive temporo-limbic patterns. The Yeo-network summaries were derived from symptom-correlated AAL regions, not from the voxel-wise group-contrast clusters. None of the regional correlations survived correction for multiple comparisons.

**Conclusion:**

The findings identify exploratory metabolic and network-level patterns associated with depressive symptoms and sleep disturbances in PD. The symptom-correlated regional distributions are compatible with, but do not validate, aspects of the body-first/brain-first framework. Larger independent cohorts, conventional voxel-wise thresholding, multiplicity-controlled analyses, and explicit adjustment for motor severity are required before symptom-specific mechanisms or PD subtypes can be inferred.

## 1 Background

Parkinson’s Disease (PD), the second most common progressive neurodegenerative disorder worldwide, is increasing in prevalence globally, placing a substantial economic and healthcare burden on patients, families, and society (Ben-Shlomo et al., 2024; Bloem et al., 2021). The pathophysiology of PD is not limited to the selective loss of dopaminergic (DA) neurons in the substantia nigra; rather, it encompasses a highly heterogeneous, multi-system disease characterized by widespread neuropathological changes across multiple brain regions and the involvement of non-dopaminergic neurons (Morris et al., 2024). This complexity results in diverse clinical manifestations, presenting significant challenges for diagnosis and treatment (Hill et al., 2023; Tinazzi et al., 2025).

Non-motor symptoms (NMS) are prevalent in PD patients and significantly impair their health-related quality of life (Leite Silva et al., 2023). These NMS, such as anxiety, depression, sleep disturbances, pain, and cognitive impairment, can precede the onset of classic motor symptoms, suggesting their potential as clinical biomarkers for early PD diagnosis and monitoring disease progression (Ahmad et al., 2023; Costa et al., 2023; Jerčić et al., 2024). Depression and sleep disturbances are among the most common NMS in PD patients, severely affecting their mental health and daily function, and are strongly associated with rapid disease progression and a poorer prognosis (Ahmad et al., 2023; Zhang et al., 2023). However, the metabolic correlates underlying PD with depression (PD-Dep) and PD with sleep disturbances (PD-SD), particularly their specific metabolic characteristics at the brain level, remain poorly understood.

Positron Emission Tomography (PET), particularly 18F-fluorodeoxyglucose (18F-FDG) PET metabolic imaging, allows for the non-invasive quantification of cerebral glucose metabolism, thereby sensitively reflecting neuronal synaptic activity and function (Meles et al., 2021; Nerattini et al., 2025). This technique has proven remarkably useful in detecting early pathological changes and identifying specific patterns of cerebral hypometabolism in neurodegenerative diseases, making it a valuable tool for early PD assessment and exploring disease mechanisms (Endo et al., 2024; Laurencin et al., 2024).

Using standardized clinical assessments and PET brain metabolic imaging, this study investigated the associations among gait impairment, depression, and sleep disturbances in Parkinson’s disease (PD) patients. Regional metabolic alterations were mapped onto large-scale brain networks to explore metabolic and network-level patterns associated with depressive symptoms (PD-Dep) and sleep disturbances (PD-SD) in two overlapping, symptom-defined PD groups. By integrating regional metabolism with network-level functional architecture, we aimed to characterize the neural correlates of these symptom profiles and generate hypotheses for future validation, without assuming that they represent distinct PD subtypes.

## 2 Subjects and methods

### 2.1 Experimental subjects

A total of 28 patients diagnosed with Parkinson’s disease (PD) at the Neurology Outpatient Department and Inpatient Ward of Inner Mongolia Medical University Affiliated Hospital between December 2019 and January 2024 were selected as the case group (age range: 50–80 years, disease duration: 0.5–8 years). All patients met the PD diagnostic criteria established by the UK Parkinson’s Disease Society Brain Bank Clinical Diagnostic Criteria (Gelb et al., 1999). Based on inclusion and exclusion criteria, PD patients were further categorized into a PD with depression group (PD-Dep, *n* = 16) and a PD with sleep disturbances, group (PD-SD, *n* = 21). During the same period, 17 healthy individuals undergoing physical examinations were recruited as the healthy control group (HC). All participants provided informed consent.

#### 2.1.1 Inclusion criteria for PD patients

(1) All patients met the PD diagnostic criteria established by the UK Parkinson’s Disease Society Brain Bank Clinical Diagnostic Criteria.

(2) Good mental status, willing and able to cooperate with relevant scale assessments and PET/CT examinations.

(3) No history of severe neurological or psychiatric disorders, no use of antidepressants, other antipsychotic medications, or long-term use of sleep-inducing drugs.

(4) Patients and/or their families provided informed consent for this study and signed the informed consent form.

#### 2.1.2 Exclusion criteria for PD patients

(1) History of deep brain stimulation (DBS) surgery.

(2) Secondary parkinsonism caused by cerebral infection, cerebrovascular disease, drugs, trauma, poisoning, tumors, or other genetic degenerative diseases.

(3) Diagnosis of Parkinson-plus syndromes, such as corticobasal degeneration, progressive supranuclear palsy, Lewy body dementia, or multiple system atrophy.

(4) Diagnosis of essential tremor, essential tremor plus syndrome, or hyperthyroidism.

(5) Presence of significant cognitive impairment (MMSE < 24).

#### 2.1.3 Inclusion criteria for HC group

(1) Age-matched with the case group.

(2) Routine physical examination showed no significant abnormalities; normal brain magnetic resonance imaging (MRI).

(3) No history of central nervous system diseases; no history of long-term use of drugs affecting the nervous system.

#### 2.1.4 Exclusion criteria for HC group

(1) Presence of significant cognitive impairment (MMSE < 24), sleep disturbances, (PSQI ≥ 7), or depressive state (HAMD ≥ 8).

(2) Inability to cooperate with relevant scale assessments and PET/CT examinations.

### 2.2 Research methods

General demographic data were collected in a one-on-one manner in the neurology outpatient department or inpatient ward. Scales were used to assess the motor, depressive, and sleep status of PD patients, as well as the depressive and sleep status of the HC group. General data included: age, sex, disease duration, etc. Assessment scales included: Modified Hoehn-Yahr (H-Y) staging scale, Unified Parkinson’s Disease Rating Scale Part III (UPDRS III), Hamilton Depression Rating Scale (HAMD), and Pittsburgh Sleep Quality Index (PSQI). Patients with HAMD scores ≥ 8 were classified as PD-Dep, and those with PSQI scores ≥ 7 as PD-SD. These groups were established based on symptom scale thresholds rather than formal psychiatric or clinical diagnoses. Nine patients met both criteria; the mutually exclusive PD categories comprised 7 PD-Dep-only, 12 PD-SD-only. In addition, the study recruited 17 healthy older adults matched in age and sex as the healthy control (HC) group. All participants underwent 18F-FDG PET-CT brain glucose metabolism imaging in the Department of Nuclear Medicine. The study protocol was reviewed and approved by the Ethics Committee of the Affiliated Hospital of Inner Mongolia Medical University (Approval Number: KY2025025).

#### 2.2.1 Scale assessments

##### 2.2.1.1 Modified Hoehn-Yahr (H-Y) Staging Scale

This scale includes eight stages ranging from 0 to 5. Stage 0 indicates no symptoms or signs; Stage 1, unilateral involvement; Stage 1.5, unilateral involvement with axial involvement; Stage 2, bilateral involvement without balance impairment; Stage 2.5, mild bilateral involvement with recovery on the pull test; Stage 3, mild-to-moderate bilateral involvement with postural instability but preserved independent living; Stage 4, severe disability with the ability to stand or walk unassisted; and Stage 5, confinement to a wheelchair or bed unless aided. Stages 1.0–2.0 were classified as early stage, 2.5–3.0 as middle stage, and 4.0–5.0 as late stage. Assessments were performed by trained neurologists.

##### 2.2.1.2 Unified Parkinson’s Disease Rating Scale Part III

The UPDRS (Unified Parkinson’s Disease Rating Scale) is a comprehensive scale for assessing the condition of PD patients. It comprises four parts: mental status, impact on activities of daily living, motor symptom assessment, and complications of therapy. In this study, we utilized UPDRS III to assess the type and severity of motor impairment in PD patients. UPDRS III primarily evaluates aspects such as speech expression, facial rigidity, resting tremor, limb and neck rigidity, postural or action tremor of the hands, dexterity of hands and legs, difficulty in rising, posture and stability, gait, and bradykinesia. Assessments were performed by trained neurologists.

##### 2.2.1.3 Hamilton Depression Rating Scale

The HAMD (Hamilton Depression Rating Scale) is currently the most commonly used clinical tool for evaluating depressive states, possessing high reliability, validity, and practicality. The HAMD consists of 24 items, including: depressed mood, feelings of guilt, suicidal ideation, insomnia (early, middle, late), work and activities, retardation, agitation, psychic anxiety, somatic anxiety, gastrointestinal symptoms, general somatic symptoms, hypochondriasis, weight loss, insight, diurnal variation, depersonalization/derealization, paranoid symptoms, feelings of helplessness, hopelessness, and worthlessness. The sum of scores for these 24 items constitutes the total HAMD score.

A total score < 8 indicates the absence of depressive symptoms; 8–20 indicates mild-to-moderate depressive symptoms; 21–34 indicates moderate-to-severe depressive symptoms; and ≥ 35 indicates severe depressive symptoms. Assessments were performed by trained psychiatrists or neurologists (Zimmerman et al., 2013).

##### 2.2.1.4 Pittsburgh Sleep Quality Index

The Pittsburgh Sleep Quality Index (PSQI) assesses sleep quality during the preceding month. It comprises 19 self-rated items, of which 18 contribute to seven component scores, plus five partner/roommate-rated items that are not included in the global score. The seven components are subjective sleep quality, sleep latency, sleep duration, habitual sleep efficiency, sleep disturbances, use of sleeping medication, and daytime dysfunction. Each component is scored from 0 to 3, yielding a global score from 0 to 21; higher scores indicate poorer sleep quality. Consistent with the prespecified operational definition used in this study, a global PSQI score ≥ 7 defined the PD-SD group. This threshold denotes clinically relevant poor subjective sleep quality rather than a formal sleep-disorder diagnosis. Assessments were performed by trained neurologists (Buysse et al., 1989).

#### 2.2.2 18 F-FDG PET brain glucose metabolism imaging

Brain 18F-FDG PET was acquired on an integrated Siemens PET/CT system whose CT component had 64-slice capability. The exact PET model identifier was not retained in the archived study records. PD medications (including levodopa, dopamine agonists, amantadine, tolcapone, and trihexyphenidyl) were withheld for at least 12 h before imaging; this interval may not have produced a fully comparable OFF state. Participants fasted for at least 6 h. Prescan glucose was required to be 3.9–6.1 mmoL/L for participants without diabetes and ≤ 11.1 mmoL/L for those with diabetes. After intravenous administration of 185 MBq 18F-FDG and 45–50 min of quiet, dark-room uptake, a CT scan was followed by one 5-min static brain PET frame. CT settings were 120 kVp, 350 effective mAs, and 3-mm slice thickness. PET data were attenuation-corrected and reconstructed using the TrueX+TOF (ultraHD-PET) method. Matrix size, reconstructed voxel dimensions, and iteration/subset settings could not be recovered from the retrospective archive and are acknowledged as a reproducibility limitation.

### 2.3 Image processing

The imaging workflow comprised two separate stages. First, PET images were realigned, their origin was reset to the anterior commissure, images were spatially normalized to MNI space, and an 8-mm Gaussian kernel was applied in SPM12 running under MATLAB 2018b (Ashburner and Friston, 2005). Voxel-wise SUVR maps were then calculated by dividing each smoothed voxel value by the mean uptake in cerebellar gray matter (Dukart et al., 2010). Separate two-sample t contrasts compared PD-Dep with HC and PD-SD with HC. The cluster-forming height threshold was *P* < 0.05 uncorrected (approximately T = 1.64); no additional minimum cluster-extent threshold was imposed; cluster-level family-wise error correction was *P* < 0.05. Because this liberal cluster-forming threshold may yield overly extensive clusters, the voxel-wise maps are reported as exploratory spatial descriptions rather than confirmatory localization. Second, regional SUVR values were extracted with PETPVE12 (Marquie et al., 2017) using the AAL atlas (Tzourio-Mazoyer et al., 2002) for exploratory correlation analyses with clinical scores. The AAL regions used for symptom-correlation network mapping were not defined by the voxel-wise group-contrast clusters.

### 2.4 Statistical analysis

Statistical analyses were performed using IBM SPSS Statistics 25.0 and Python 3.9. Continuous variables are presented as mean ± SD with 95% CI or median (P25, P75), and categorical variables as n (%). Because nine patients overlapped between PD-Dep and PD-SD, these groups were not compared directly; each was compared with HC. Age was analyzed using independent-samples *t*-tests; HAMD, PSQI, and prescan glucose using Mann–Whitney U tests; and sex and diabetes using Fisher’s exact tests. The two *P*-values for each variable were adjusted by the Holm method. Partial correlations in PD-Dep were adjusted for age, sex, disease duration, and PSQI, whereas those in PD-SD were adjusted for age, sex, disease duration, and HAMD. UPDRS-III was not added to these symptom models because the small group sizes and overlap among clinical covariates would further reduce residual degrees of freedom and produce unstable estimates. Consequently, symptom specificity was not established. Correlation *P*-values are nominal and none survived multiplicity correction.

## 3 Results

### 3.1 General characteristics

Table 1 summarizes the demographic and clinical characteristics of the symptom-defined PD groups and healthy controls. Neither age nor sex differed significantly between PD-Dep and HC or between PD-SD and HC (all Holm-adjusted *P* > 0.05). HAMD and PSQI scores were higher in both PD groups than in HC (all Holm-adjusted *P* < 0.001). Pre-scan blood glucose was 5.30 (5.10, 5.80) mmoL/L in PD-Dep, 5.50 (5.10, 5.80) mmoL/L in PD-SD, and 5.20 (4.90, 5.60) mmoL/L in HC; neither comparison with HC was significant (P_1_ = 0.552 and P_2_ = 0.552). Diabetes was present in 3/16 (18.8%) PD-Dep participants, 3/21 (14.3%) PD-SD participants, and 1/17 (5.9%) HC participants, with no significant difference in either comparison (P_1_ = 0.671 and P_2_ = 0.671). Disease duration, H–Y stage, and UPDRS-III score are reported descriptively for the PD groups only.

**TABLE 1:** Demographic characteristics of PD-Dep, PD-SD, and healthy control groups.

Variables	PD-Dep (*n* = 16)	PD-SD (*n* = 21)	HC (*n* = 17)	P1	P2
Sex (male), n (%)	8 (50.0)	9 (42.9)	8 (47.1)	1.000	1.000
Diabetes mellitus, n (%)	3 (18.8)	3 (14.3)	1 (5.9)	0.671	0.671
Age (years), mean ± SD (95% CI)	66.31 ± 6.65 (62.77–69.86)	66.19 ± 5.66 (63.61–68.77)	64.29 ± 6.20 (61.11–67.48)	0.664	0.664
Disease duration (years), mean ± SD (95% CI)	4.00 ± 1.97 (2.95–5.05)	3.60 ± 1.71 (2.81–4.38)	–	–	–
H–Y score, mean ± SD (95% CI)	3.09 ± 1.05 (2.53–3.65)	2.67 ± 1.09 (2.17–3.16)	–	–	–
UPDRS-III score, mean ± SD (95% CI)	37.50 ± 16.67 (28.62–46.38)	36.76 ± 17.55 (28.77–44.75)	–	–	–
HAMD score, median (P25, P75)	15.50 (12.75, 20.50)	14.00 (7.00, 20.00)	4.00 (3.00, 5.00)	< 0.001	<0.001
PSQI score, median (P25, P75)	12.00 (8.75, 15.00)	12.00 (11.00, 14.00)	4.00 (4.00, 5.00)	< 0.001	<0.001
Pre-scan blood glucose (mmoL/L), median (P25, P75)	5.30 (5.10, 5.80)	5.50 (5.10, 5.80)	5.20 (4.90, 5.60)	0.552	0.552

### 3.2 Comparison of SUVR in various brain regions between the PD-Dep and HC groups

**FIGURE 1:**
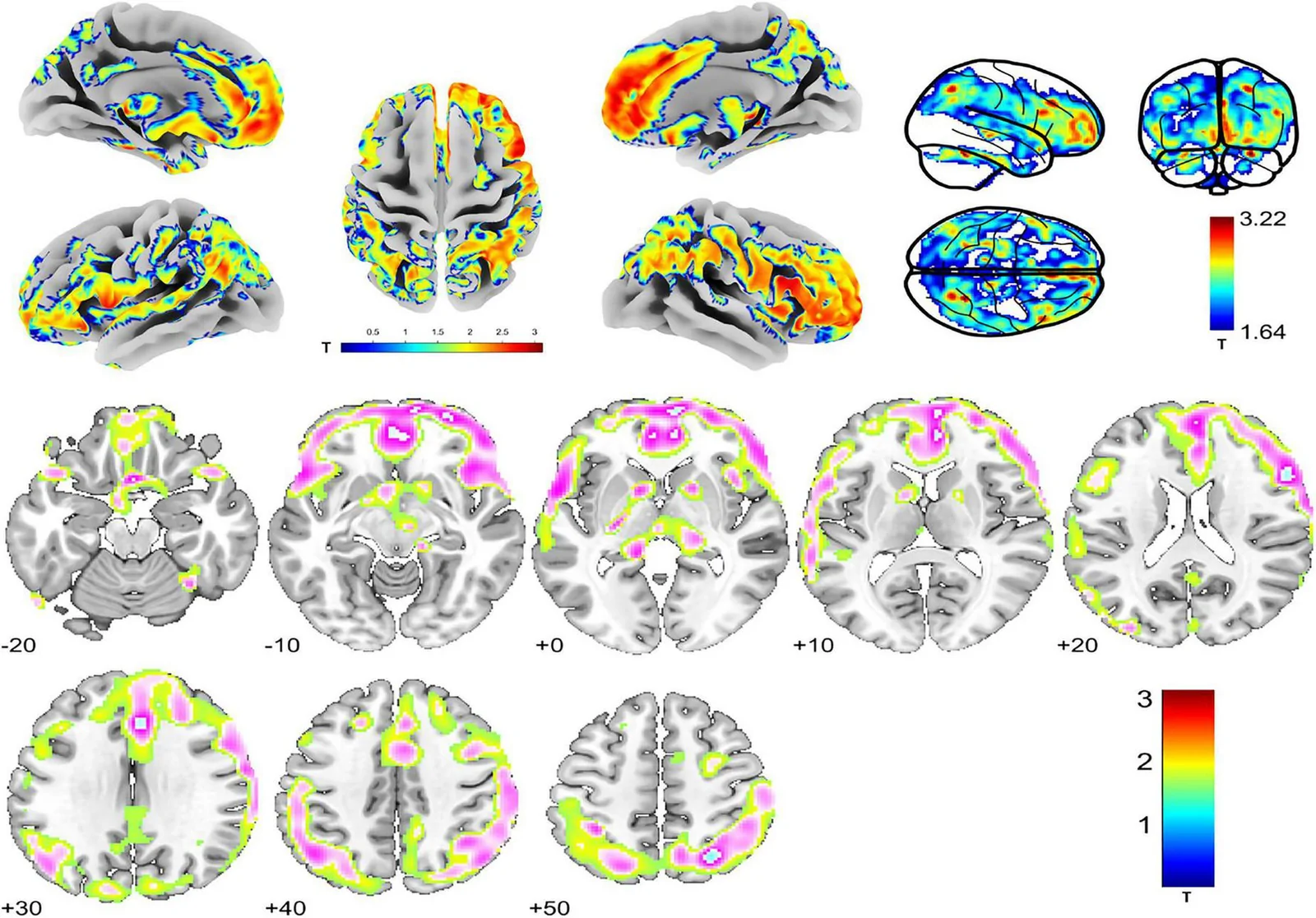
Exploratory PD-Dep versus HC voxel-wise contrast. The map shows bilateral frontal, parietal, temporal, and occipital SUVR reductions, with the largest cluster in the right hemisphere. Contrast direction: PD-Dep < HC. Cluster-forming height threshold: *P* < 0.05 uncorrected (approximately *T* = 1.64). Minimum cluster extent: none imposed. Correction method: cluster-level FWE *P* < 0.05. Because the cluster-forming threshold was liberal, the spatial extent should be interpreted cautiously. Orientations correspond to anatomical left and right without mirror reversal.

At the prespecified exploratory threshold, the PD-Dep versus HC contrast showed bilateral SUVR reductions involving frontal, parietal, temporal, and occipital regions (Figure 1 and Table 2). The largest cluster was in the right hemisphere (11,183 voxels), with additional right temporal and left frontotemporal and parietal clusters. Because the liberal cluster-forming threshold generated large spatially extensive clusters, these maps should not be interpreted as anatomically specific confirmatory findings.

**TABLE 2:** Brain regions with reduced metabolism in the PD-Dep group compared to the HC group.

Hemisphere	Cluster	Number of voxels	Peak MNI coordinate	Peak intensity	*P*	Voxels	Structure
R	Cluster1	11,183	4, 26, 34	3.146	0.002		
						1,921	Medial frontal gyrus
						1,775	Inferior frontal gyrus
						1,296	Limbic lobe
						1,222	Superior frontal gyrus
						1,142	Middle frontal gyrus
						955	Anterior cingulate
						511	Inferior parietal lobule
						495	Angular gyrus
						443	Superior parietal lobule
						440	Supramarginal gyrus
						417	Precuneus
						399	Cingulate gyrus
						167	Temporal lobe
R	Cluster2	1,394	32, −52, −28	3.193	0.002		
						1,193	Temporal lobe
						115	Fusiform gyrus
						86	Parahippocampa gyrus
L	Cluster3	6,608	−58, 8, 2	2.508	0.009		
						1,505	Superior temporal gyrus
						1,871	Middle frontal gyrus
						1,377	Inferior frontal gyrus
						857	Inferior temporal gyrus
						673	Middle temporal gyrus
						325	Transverse temporal gyrus
L	Cluster4	2,837	−46, −64, 36	2.493	0.009		
						1,453	Postcentral gyrus
						375	Inferior parietal lobule
						220	Angular gyrus
						194	Superior parietal lobule
						183	Precuneus
						132	Temporal lobe
						111	Supramarginal gyrus
						102	Middle temporal gyrus
						55	Middle occipital gyrus
						12	Superior temporal gyrus

### 3.3 Comparison of SUVR between the PD-SD and HC groups

**FIGURE 2:**
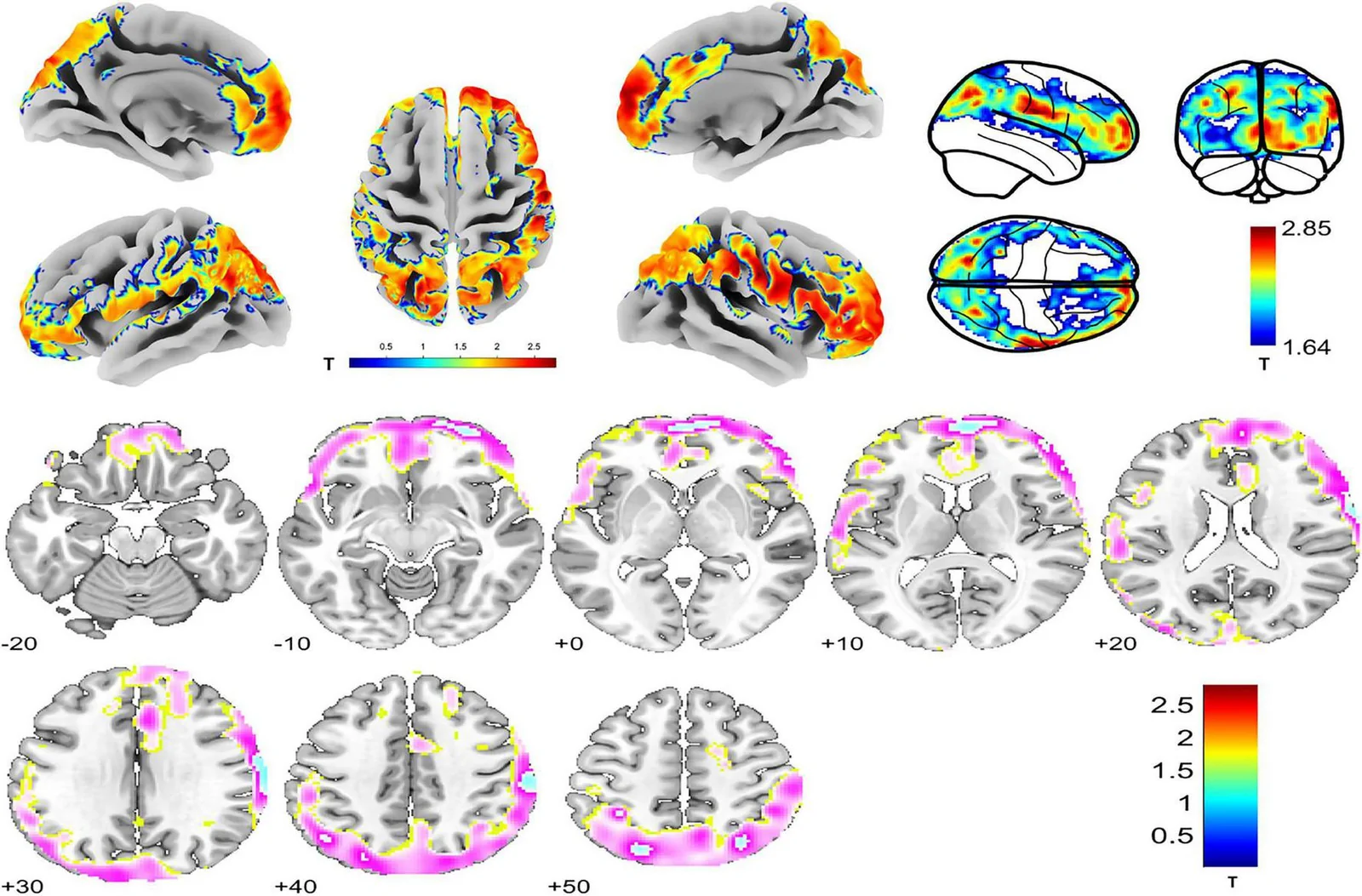
Exploratory PD-SD versus HC voxel-wise contrast. The map shows bilateral frontal, parietal, temporal, occipital, and cingulate SUVR reductions. Contrast direction: PD-SD < HC. Cluster-forming height threshold: *P* < 0.05 uncorrected (approximately *T* = 1.64). Minimum cluster extent: none imposed. Correction method: cluster-level FWE *P* < 0.05. Because the cluster-forming threshold was liberal, the spatial extent should be interpreted cautiously. Orientations correspond to anatomical left and right without mirror reversal.

At the same exploratory threshold, the PD-SD versus HC contrast showed bilateral SUVR reductions in frontal, parietal, temporal, occipital, and cingulate regions (Figure 2 and Table 3). The largest reported clusters were spatially extensive. These voxel-wise results are therefore presented as exploratory spatial patterns and not as definitive anatomical localization.

**TABLE 3:** Distribution of brain regions with reduced metabolism in the PD-SD group compared to the HC group.

Hemisphere	Cluster	Number of voxels	Peak MNI coordinate	Peak intensity	*P*	Voxels	Structure
R	Cluster1	5,766	68, −14, 28	2.6614	0.006		
						1,105	Superior frontal gyrus
						1,026	Medial frontal gyrus
						967	Inferior frontal gyrus
						816	Superior parietal lobe
						554	Supramarginal gyrus
						520	Postcentral gyrus
						390	Inferior parietal lobule
						330	Angular gyrus
						58	Anterior cingulate
R	Cluster2	318	4, 26, 32	2.1832	0.018		
						118	Limbic lobe
						178	Middle cingulate gyrus
						22	Anterior cingulate
L	Cluster3	454	−64, −16, 14	1.9992	0.026		
						148	Postcentral gyrus
						67	Temporal lobe
						60	Supramarginal gyrus
						72	Superior temporal gyrus
						44	Transverse temporal gyrus
						32	Precentral gyrus
						31	Inferior parietal lobule
L	Cluster4	8,683	−26, −72, 46	2.523	0.008		
						1,938	Inferior frontal gyrus
						1,854	Superior frontal gyrus
						1,157	Middle frontal gyrus
						1,011	Precuneus
						813	Superior parietal lobule
						521	Cuneus
						374	Inferior parietal lobule
						372	Angular gyrus
						335	Superior occipital gyrus
						236	Middle occipital gyrus
						72	Supramarginal gyrus

The voxel-wise contrasts suggested partially overlapping frontoparietal patterns in the two symptom-defined PD cohorts. Given the overlapping membership and liberal cluster-forming threshold, the contrasts do not establish unique or symptom-specific metabolic signatures.

## 4 Discussion

### 4.1 Metabolic correlates of depressive symptoms and motor severity in PD-Dep

#### 4.1.1 Correlation between regional brain metabolism and HAMD scores in the PD-Dep group

Exploratory heatmaps and scatter plots showed nominal associations between regional SUVR and depressive or motor scores in PD-Dep. These AAL-based correlations were analytically separate from the voxel-wise PD-Dep versus HC contrast.

**FIGURE 3:**
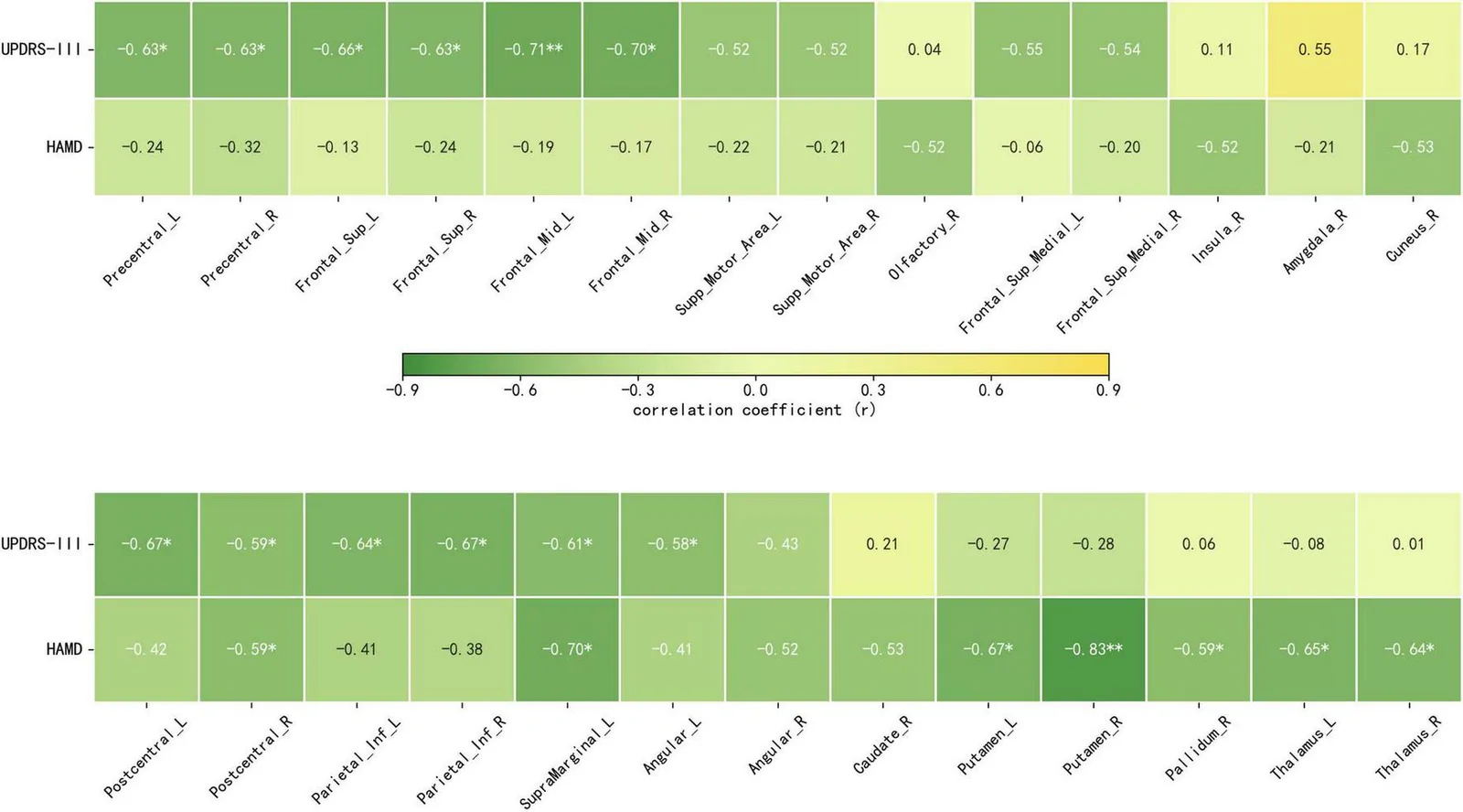
Exploratory partial correlations between regional SUVR and HAMD or UPDRS-III in PD-Dep. Yellow and green indicate positive and negative correlations, respectively. HAMD models were adjusted for age, sex, disease duration, and PSQI; UPDRS-III was not included in the HAMD model. |*r*| > 0.5; * nominal *P* < 0.05; ** nominal *P* < 0.01. None survived multiplicity correction.

**FIGURE 4:**
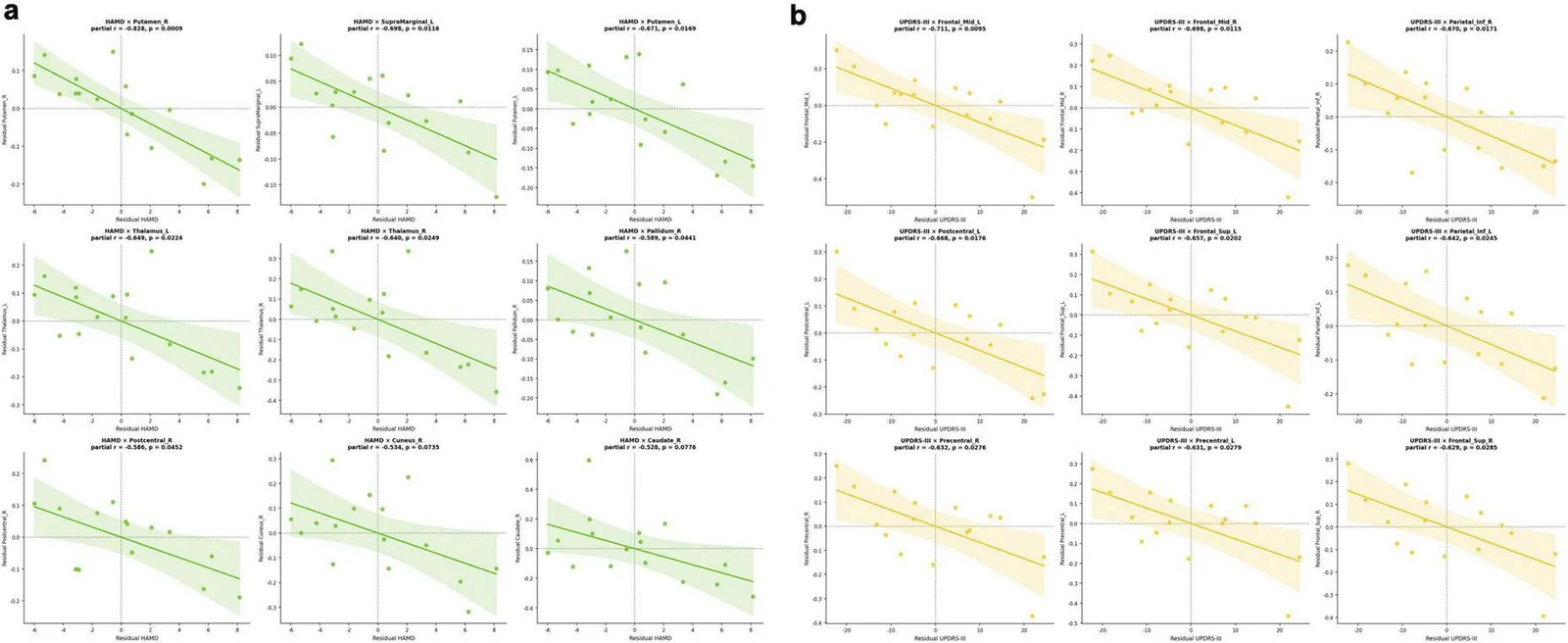
**(a)** Scatter plots of the partial correlation between regional SUVR and HAMD scores in the PD-Dep group. **(b)** Scatter plots of the partial correlation between regional SUVR and UPDRS-III scores in the PD-Dep group.

Among the 27 AAL regions examined for the HAMD analysis, seven showed nominal *P* < 0.05 and |r| > 0.5 (Figures 3, 4a, Supplementary Figure 1 and Supplementary Table 1). These included the bilateral putamen, bilateral thalamus, right pallidum, left supramarginal gyrus, and right postcentral gyrus. The putamen, pallidum, and thalamus were identified only through the symptom-correlation analysis; they were not components of the PD-Dep versus HC hypometabolism clusters in Table 2 and therefore are not described as a group-level hypometabolic signature. Nevertheless, accumulating evidence highlights the critical involvement of these structures: in PD with depression, glucose metabolism within this circuit—particularly in the pallidum and thalamus—is significantly reduced, suggesting impaired motor-emotion integration and representing a key neuroimaging mechanism of PD depression (Chen et al., 2023; Hou et al., 2026). Specifically, Chen et al. (2023) confirmed that basal ganglia-thalamocortical circuit connectivity is impaired in PD, with functional connectivity negatively correlated with depression scores, which aligns well with our observed CSPT-HAMD coupling. Furthermore, Hou et al., 2026) demonstrated that PD depression involves widespread alterations across basal ganglia, thalamocortical circuits, and multiple neurotransmitter systems. Although these nominal associations are anatomically compatible with CSPT involvement and echo these recent findings, none survived correction for multiple comparisons and UPDRS-III was not included as a covariate. They should therefore be treated as hypothesis-generating associations rather than definitive evidence of a depression-specific mechanism.

#### 4.1.2 Correlation between regional brain metabolism and UPDRS-III scores in the PD-Dep group

For UPDRS-III, 12 frontoparietal AAL regions showed nominal *P* < 0.05 and |r| > 0.5 (Figures 3, 4b, Supplementary Figure 2 and Supplementary Table 1), including bilateral middle frontal gyri and inferior parietal lobules. This spatial convergence is strongly corroborated by recent neuroimaging studies: Sigurdsson et al. (2022) constructed gait-related metabolic covariance networks using resting-state FDG-PET and similarly found that the middle frontal gyrus and inferior parietal lobule constitute core nodes of gait-cognitive metabolic covariance. Furthermore, Wu et al. (2023) used integrated PET/MRI to confirm that frontoparietal network metabolic reduction is significantly associated with motor impairment severity in PD, while Li et al. (2025) further revealed that the metabolic-functional network characteristics of these regions serve as key imaging biomarkers for PD. The significant hypometabolism in these frontoparietal regions observed in our PD-Dep patients thus aligns well with these findings. However, because of the overlap between these motor associations and the HAMD-associated cortical regions, it indicates that general motor severity may contribute to the observed symptom–metabolism relationships. Accordingly, no symptom-specific interpretation is made.

### 4.2 Metabolic correlates of sleep quality and motor severity in PD-SD

#### 4.2.1 Correlation between regional brain metabolism and PSQI scores in the PD-SD group

**FIGURE 5:**
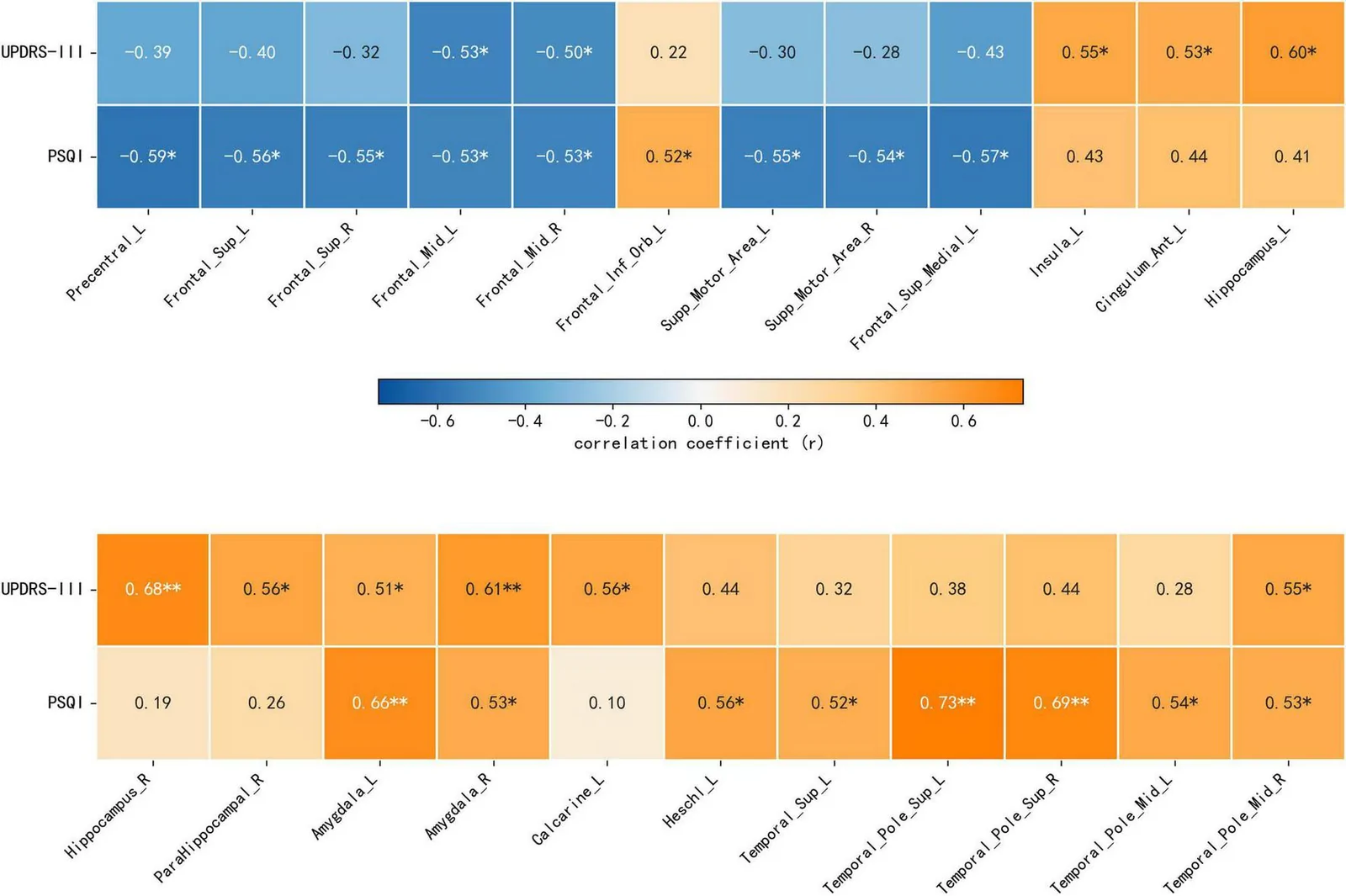
Exploratory partial correlations between regional SUVR and PSQI or UPDRS-III in the PD-SD group. Orange and blue indicate positive and negative correlations, respectively. PSQI models were adjusted for age, sex, disease duration, and HAMD; UPDRS-III was not included in the PSQI model. |*r*| > 0.5; * nominal *P* < 0.05; ** nominal *P* < 0.01. None survived multiplicity correction.

Figure 5 shows that regional SUVRs are differentially associated with sleep quality and motor symptoms, as detailed below.

**FIGURE 6:**
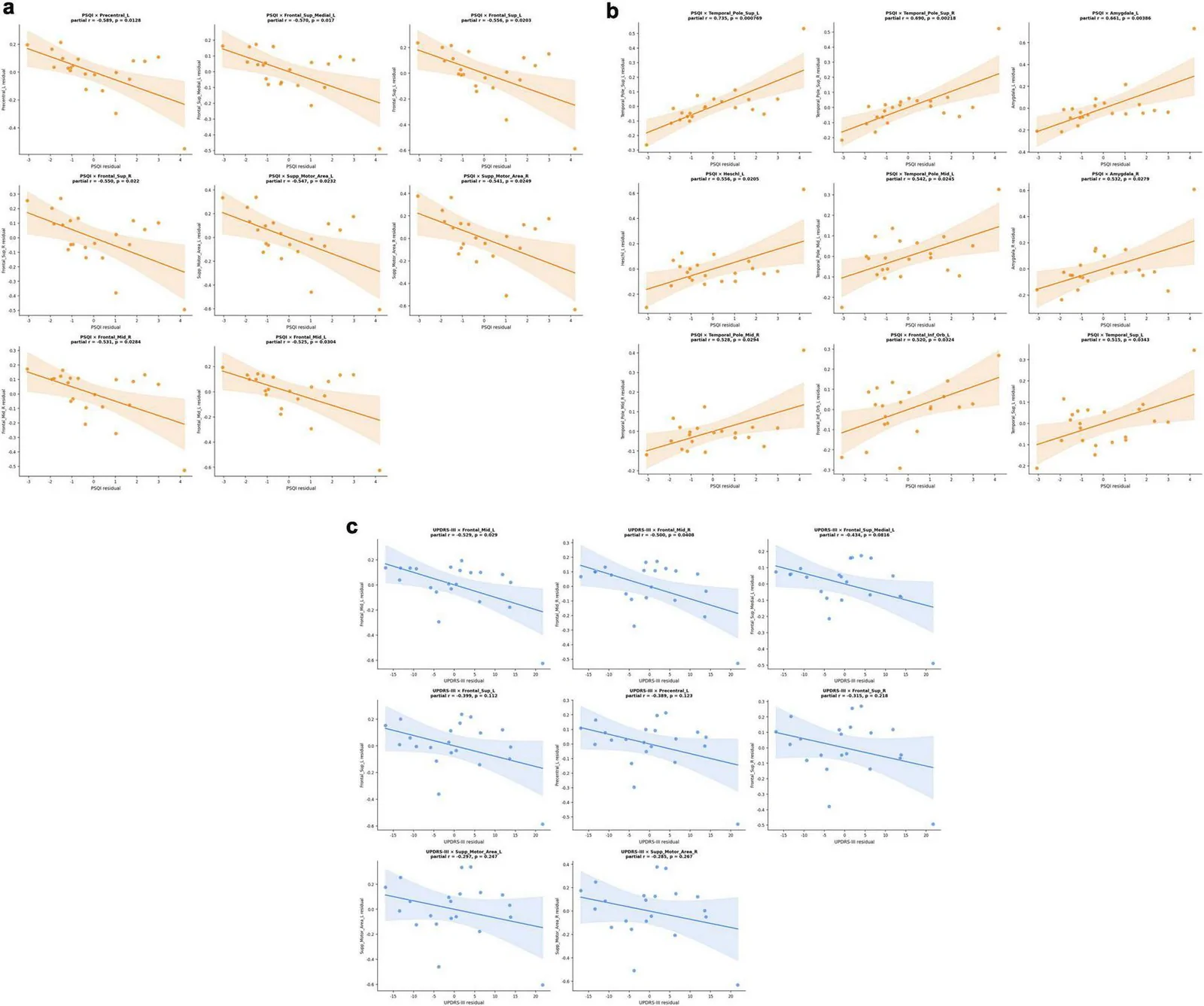
**(a)** Scatter plots of the partial correlation between regional SUVR and PSQI scores in the PD-SD group (negative correlation). **(b)** Scatter plots of the partial correlation between regional SUVR and PSQI scores in the PD-SD group (positive correlation). **(c)** Scatter plots of the partial correlation between regional SUVR and UPDRS-III scores in the PD-SD group.

The exploratory PD-SD heatmap and scatter plots (Figures 5, 6a,b, Supplementary Figure 3 and Supplementary Table 2) showed nominal negative and positive associations between regional SUVR and PSQI. Twenty-three AAL regions met the descriptive |r| > 0.5 criterion, of which 17 had nominal *P* < 0.05; none survived multiplicity correction.

Nominal negative PSQI associations were concentrated in frontal and somatomotor regions, whereas nominal positive associations involved temporo-limbic regions. Because PD-SD was defined solely by PSQI and neither polysomnography nor a validated RBD instrument was available, these observations cannot be interpreted as evidence for RBD disinhibition, RBD phenoconversion, or a specific sleep pathophysiology. They are retained only as hypothesis-generating regional associations.

#### 4.2.2 Correlation between regional brain metabolism and UPDRS-III scores in the PD-SD group

For UPDRS-III in PD-SD, 11 of the 23 AAL regions showed nominal *P* < 0.05 (Figures 5, 6c, Supplementary Figure 4 and Supplementary Table 2), including positive associations in limbic and occipital regions and negative associations in the bilateral middle frontal gyri. These findings did not survive multiplicity correction and should not be interpreted as pathological overactivation, compensation, RBD-related change, or a sleep-specific mechanism.

### 4.3 Exploratory Yeo 7 network mapping of symptom-correlated AAL regions

For descriptive network visualization, the 27 PD-Dep and 23 PD-SD AAL regions (Tzourio-Mazoyer et al., 2002) included in the exploratory symptom-correlation analyses were mapped onto the Yeo 7 functional network atlas (Yeo et al., 2011). These regions were selected from the correlation workflow and were not derived from, or required to overlap with, the voxel-wise PD-versus-HC group contrasts in Tables 2, 3. Accordingly, the following network counts describe the distribution of symptom-correlated regions, not hypometabolic group-difference signatures.

#### 4.3.1 Network distribution of PD-Dep symptom-correlated regions

**FIGURE 7:**
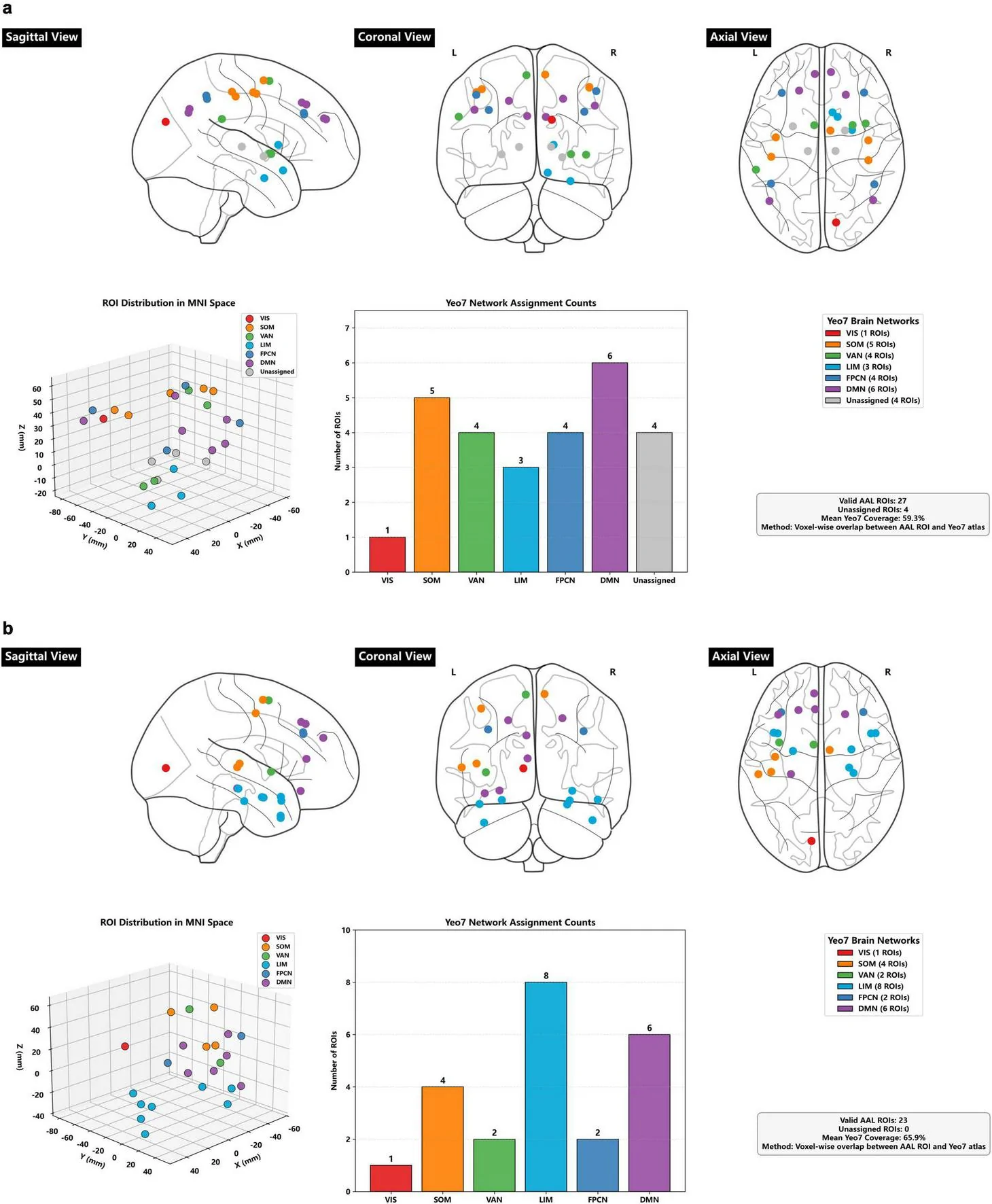
**(a)** Descriptive Yeo 7 mapping of 27 AAL regions included in the exploratory PD-Dep symptom-correlation analyses. Node colors indicate assigned networks; subcortical regions outside the cortical Yeo template are shown separately. These regions were not selected from the PD-Dep versus HC voxel-wise contrast and should not be interpreted as a group-level hypometabolic signature. **(b)** Descriptive Yeo 7 mapping of 23 AAL regions included in the exploratory PD-SD symptom-correlation analyses. Node colors indicate assigned networks. These regions were not selected from the PD-SD versus HC voxel-wise contrast and should not be interpreted as a group-level hypometabolic signature.

For PD-Dep, the 27 symptom-correlated AAL regions were distributed across the DMN (6 regions, 22.2%), SOM (5 regions), FPCN (4 regions), VAN (4 regions), LIM (3 regions), and VIS (1 region), while four subcortical regions fell outside the Yeo cortical template (Figure 7a). Specifically, the DMN was the most extensively affected network—comprising the bilateral angular gyri, bilateral superior frontal gyri, and bilateral medial superior frontal gyri—which dominates self-referential processing, autobiographical memory retrieval, and introspective thinking (Lin et al., 2026; She et al., 2025). The SOM accounted for 5 regions (bilateral precentral gyri, right SMA, and bilateral postcentral gyri), responsible for motor execution, somatosensory feedback integration, and action planning (Sigurdsson et al., 2022). The FPCN comprised 4 regions (bilateral middle frontal gyri and bilateral inferior parietal lobules), involved in cognitive control, working memory maintenance, and flexible task switching (Johansson et al., 2022; Wang et al., 2021). The VAN included 4 regions (left SMA, right insula, left supramarginal gyrus, and right putamen), mediating salience detection, bottom-up attention orienting, and stimulus-driven behavioral redirection (Jonkman et al., 2021; Wang et al., 2021). The LIM contained 3 regions (right olfactory cortex, right amygdala, and right caudate), engaging in emotional processing, reward evaluation, and motivational regulation (Baagil et al., 2023; Togo et al., 2023), whereas the VIS had only 1 region (right cuneus), responsible for primary visual processing and visuospatial integration (Baun et al., 2024).

Additionally, the four subcortical regions—the left putamen, right pallidum, and bilateral thalamus—entered this map through nominal symptom correlations and were not hypometabolic regions in the PD-Dep versus HC contrast. These subcortical structures belong to the CSPT complex, the core pathway regulating motor initiation, coordination, and sensory gating (Chen et al., 2023; Sumarac et al., 2024). Notably, the prominent involvement of the DMN (22.2%) aligns closely with recent literature highlighting DMN and salience network dysfunction in PD depression (She et al., 2025), as well as evidence demonstrating that disrupted DMN integration correlates with HAMD scores (Lin et al., 2026). Nevertheless, these network proportions remain strictly descriptive and should not be interpreted as establishing a PD-Dep-specific CSPT signature.

#### 4.3.2 Network distribution of PD-SD symptom-correlated regions

For PD-SD, the 23 symptom-correlated AAL regions were distributed across the LIM (8 regions, 34.8%: bilateral amygdalae, bilateral superior and middle temporal poles, right hippocampus, and right parahippocampal gyrus), DMN (6 regions), SOM (4 regions), VAN (2 regions), FPCN (2 regions), and VIS (1 region), with a complete absence of the subcortical CSPT complex (Figure 7b). Compared to PD-Dep, where the LIM accounted for only 11.1% (3 regions), this limbic predominance in PD-SD (34.8%) constitutes the most salient network-level difference between the two symptom profiles. Specifically, core limbic hubs—including the amygdala, hippocampus, and parahippocampal gyrus—exhibited significant metabolic alterations, suggesting that sleep disturbances in PD-SD extend beyond mere arousal dysregulation to involve widespread dysfunction in multiple limbic-dependent processes, such as emotional memory encoding, fear extinction learning, and sleep-wake rhythm regulation. This spatial pattern is corroborated by prior work: Xu et al. (2022) noted in a longitudinal review that PD sleep disturbances involve progressive damage to the hypothalamic-limbic-prefrontal multilevel circuit, forming a bidirectional pathological cycle between sleep fragmentation and limbic hypometabolism. Furthermore, Lin et al. (2025) revealed through multimodal MRI that PD patients with sleep/RBD comorbidities show widespread abnormalities in intra-limbic and limbic-neocortical functional connectivity, reinforcing the limbic system as a core pathological substrate.

Nevertheless, this relatively larger limbic proportion remains a descriptive feature of this selected correlation set. Because the patient group was defined by PSQI alone, the distribution cannot be attributed specifically to RBD or another objectively diagnosed sleep disorder, nor does it establish definitive limbic neurodegeneration.

#### 4.3.3 Relation to the brain-first/body-first hypothesis

The descriptive distributions of symptom-correlated regions are compatible with selected elements of the brain-first/body-first framework (Borghammer, 2023; Borghammer, 2023; Horsager et al., 2020), but they do not validate that framework or identify pathological propagation routes. In particular, the CSPT regions in PD-Dep arose from nominal symptom correlations rather than the PD-Dep versus HC contrast, and the PD-SD cohort was defined by subjective sleep quality rather than an RBD measure. The small, overlapping cohorts, lack of multiplicity-corrected regional associations, absence of UPDRS-III adjustment in the symptom models, and lack of a PD control group without depressive symptoms or sleep disturbance preclude assignment of body-first or brain-first subtypes. The present observations are therefore framed solely as hypothesis-generating patterns for testing in larger, prospectively phenotyped cohorts.

Several methodological limitations require emphasis. First, the voxel-wise maps used a liberal *P* < 0.05 uncorrected cluster-forming threshold; a conventional *P* ≤ 0.001 analysis was not performed, so spatial extent and anatomical specificity may be overestimated. Second, cerebellar gray matter normalization was not compared with global-mean normalization; reference-region choice remains an unresolved source of uncertainty, particularly near the occipital–cerebellar boundary. Third, UPDRS-III was not included in symptom partial-correlation models, and symptom specificity remains unestablished. Fourth, complete formulation-specific LEDD could not be reconstructed retrospectively, and a 12-h medication withdrawal may not eliminate residual dopaminergic or anticholinergic effects on FDG uptake. Fifth, voxel-wise group-effect sizes with confidence intervals were not available. Finally, the exact PET model and complete reconstruction settings were not retained, no individual structural MRI was available for atrophy or partial-volume assessment, and analysis code was not deposited. These limitations restrict the findings to exploratory associations.

## 5 Conclusion

This exploratory study identified voxel-wise and regional metabolic patterns associated with overlapping, scale-defined depressive symptoms and sleep disturbances in PD. The voxel-wise contrasts used a liberal cluster-forming threshold, while the Yeo network summaries were derived separately from nominal symptom-correlated AAL regions. The observed cortical, subcortical, and limbic distributions are compatible with hypotheses concerning heterogeneous PD networks but do not validate body-first/brain-first subtypes or establish symptom-specific mechanisms. Confirmation requires larger prospectively phenotyped cohorts, conventional voxel-wise thresholding, multiplicity-controlled regional analyses, adjustment for motor severity, and robustness analyses using alternative normalization strategies.

## Data Availability

The datasets generated and analyzed during the current study are not publicly available due to participant privacy, ethical restrictions, and informed consent limitations, but de-identified data may be available from the corresponding author upon reasonable request and approval. Requests to access these datasets should be directed to likaidi619@163.com.

## References

[B1] Ahmad M. H. Rizvi M. A. Ali M. Mondal A. C. (2023). Neurobiology of depression in Parkinson’s disease: Insights into epidemiology, molecular mechanisms and treatment strategies. *Ageing. Res. Rev*. 85:101840. 10.1016/j.arr.2022.101840 36603690

[B2] Ashburner J. Friston K. J. (2005). Unified segmentation. *Neuroimage* 26 839–851. 10.1016/j.neuroimage.2005.02.018 15955494

[B3] Baagil H. Hohenfeld C. Habel U. Eickhoff S. B. Gur R. E. Reetz K. et al. (2023). Neural correlates of impulse control behaviors in Parkinson’s disease: Analysis of multimodal imaging data. *Neuroimage. Clin*. 37:103315. 10.1016/j.nicl.2023.103315 36610308 PMC9850204

[B4] Baun A. M. Iranzo A. Terkelsen M. H. Stokholm M. G. Stær K. Serradell M. et al. (2024). Cuneus atrophy and Parkinsonian phenoconversion in cognitively unimpaired patients with isolated REM sleep behavior disorder. *J. Neurol*. 272:59. 10.1007/s00415-024-12762-x 39680182

[B5] Ben-Shlomo Y. Darweesh S. Llibre-Guerra J. Marras C. San Luciano M. Tanner C. (2024). The epidemiology of Parkinson’s disease. *Lancet* 403 283–292. 10.1016/S0140-6736(23)01419-8 38245248 PMC11123577

[B6] Bloem B. R. Okun M. S. Klein C. (2021). Parkinson’s disease. *Lancet* 397 2284–2303. 10.1016/S0140-6736(21)00218-X 33848468

[B7] Borghammer P. (2023). The brain-first vs. Body-first model of Parkinson’s disease with comparison to alternative models. *J. Neural. Transm*. 130 737–753. 10.1007/s00702-023-02633-6 37062013

[B8] Buysse D. J. Reynolds C. F. Monk T. H. Berman S. R. Kupfer D. J. (1989). The Pittsburgh Sleep Quality Index: a new instrument for psychiatric practice and research. *Psychiatry Res.* 28, 193–213. 10.1016/0165-1781(89)90047-4 2748771

[B9] Chen L. Sun J. Gao L. Wang J. Ma J. Xu E. et al. (2023). Dysconnectivity of the parafascicular nucleus in Parkinson’s disease: A dynamic causal modeling analysis. *Neurobiol. Dis*. 188:106335. 10.1016/j.nbd.2023.106335 37890560

[B10] Costa H. N. Esteves A. R. Empadinhas N. Cardoso S. M. (2023). Parkinson’s disease: A multisystem disorder. *Neurosci. Bull*. 39 113–124. 10.1007/s12264-022-00934-6 35994167 PMC9849652

[B11] Dukart J. Mueller K. Horstmann A. Vogt B. Frisch S. Barthel H. et al. (2010). Differential effects of global and cerebellar normalization on detection and differentiation of dementia in FDG-PET studies. *Neuroimage* 49 1490–1495. 10.1016/j.neuroimage.2009.09.017 19770055

[B12] Endo H. Ono M. Takado Y. Matsuoka K. Takahashi M. Tagai K. et al. (2024). Imaging α-synuclein pathologies in animal models and patients with Parkinson’s and related diseases. *Neuron* 112 2540e–2557e. 10.1016/j.neuron.2024.05.006 38843838

[B13] Gelb D. J. Oliver E. Gilman S. (1999). Diagnostic criteria for Parkinson disease. *Arch. Neurol*. 56 33–39. 10.1001/archneur.56.1.33 9923759

[B14] Hill D. R. Huters A. D. Towne T. B. Reddy R. E. Fogle J. L. Voight E. A. et al. (2023). Parkinson’s disease: Advances in treatment and the syntheses of various classes of pharmaceutical drug substances. *Chem. Rev*. 123 13693–13712. 10.1021/acs.chemrev.3c00479 37975808

[B15] Horsager J. Andersen K. B. Knudsen K. Skjærbæk C. Fedorova T. D. Okkels N. et al. (2020). Brain-first versus body-first Parkinson’s disease: A multimodal imaging case-control study. *Brain* 143 3077–3088. 10.1093/brain/awaa238 32830221

[B16] Hou X. Zhang J. Duan J. Song Y. Tang X. Gong Z. et al. (2026). Multimodal MRI data fusion reveals distinct structural, functional and neurochemical correlates of depression in patients with Parkinson’s disease. *Neuroimage* 327:121706. 10.1016/j.neuroimage.2026.121706 41512941

[B17] Jerčić K. G. Blažeković A. Borovečki S. Borovečki F. (2024). Non-motor symptoms of Parkinson‘s disease-insights from genetics. *J. Neural. Transm*. 131 1277–1284. 10.1007/s00702-024-02833-8 39294309

[B18] Johansson M. E. Cameron I. G. M. Van der Kolk N. M. de Vries N. M. Klimars E. Toni I. et al. (2022). Aerobic exercise alters brain function and structure in Parkinson’s disease: A randomized controlled trial. *Ann. Neurol*. 91 203–216. 10.1002/ana.26291 34951063 PMC9306840

[B19] Jonkman L. E. Fathy Y. Y. Berendse H. W. Schoonheim M. M. van de Berg W. D. J. (2021). Structural network topology and microstructural alterations of the anterior insula associate with cognitive and affective impairment in Parkinson’s disease. *Sci. Rep*. 11:16021. 10.1038/s41598-021-95638-8 34362996 PMC8346470

[B20] Laurencin C. Lancelot S. Brosse S. Mérida I. Redouté J. Greusard E. et al. (2024). Noradrenergic alterations in Parkinson’s disease: A combined 11C-yohimbine PET/neuromelanin MRI study. *Brain* 147 1377–1388. 10.1093/brain/awad338 37787503 PMC10994534

[B21] Leite Silva A. B. R. Gonçalves de Oliveira R. W. Diógenes G. P. de Castro Aguiar M. F. Sallem C. C. Lima M. P. P. et al. (2023). Premotor, nonmotor and motor symptoms of Parkinson’s disease: A new clinical state of the art. *Ageing. Res. Rev*. 84:101834. 10.1016/j.arr.2022.101834 36581178

[B22] Li X. Zhang Y. Wu J. Yao X. Zhao Z. Li R. et al. (2025). Multimodal metabolic and functional network signatures for diagnosis of Parkinson’s disease: A PET/MR study. *Sci. Rep*. 15:35685. 10.1038/s41598-025-19652-w 41083689 PMC12518659

[B23] Lin H. Cheng X. Xu Y. Wu J. Zhu J. Mao C. et al. (2025). Multimodal MRI changes associated with non-motor symptoms of rapid eye movement sleep behaviour disorder in Parkinson’s disease patients. *Neuroradiology* 67 153–162. 10.1007/s00234-024-03492-y 39476126

[B24] Lin Y. Tang Y. Tan C. Wang C. Liu Q. Shen Q. et al. (2026). Dynamic functional connectivity changes in Parkinson’s disease with and without depression. *Eur. J. Neurosci*. 63:e70549. 10.1111/ejn.70549 42163705

[B25] Marquie M. Siao M. Tick Chong A. Anton-Fernandez E. E. Verwer N. Saez-Calveras A. C. et al. (2017). [F-18]-AV-1451 binding correlates with postmortem neurofibrillary tangle braak staging. *Acta. Neuropathol.* 134 619–628. 10.1007/s00401-017-1740-8 28612291 PMC5772971

[B26] Meles S. K. Oertel W. H. Leenders K. L. (2021). Circuit imaging biomarkers in preclinical and prodromal Parkinson’s disease. *Mol. Med*. 27:111. 10.1186/s10020-021-00327-x 34530732 PMC8447708

[B27] Morris H. R. Spillantini M. G. Sue C. M. Williams-Gray C. H. (2024). The pathogenesis of Parkinson’s disease. *Lancet* 403 293–304. 10.1016/S0140-6736(23)01478-2 38245249

[B28] Nerattini M. Abenavoli E. M. Berti V. (2025). PET/CT in movement disorders: Update. *Semin Nucl Med*. 55 565–576. 10.1053/j.semnuclmed.2025.03.007 40320359

[B29] She Z. Qi X. Shi X. Peng H. Zheng J. Ma J. et al. (2025). Serum sirtuin 3 levels and multimodal abnormalities in brain structure and function in Parkinson’s disease patients with depression. *Neurol. Sci*. 46 3663–3676. 10.1007/s10072-025-08170-2 40259181 PMC12267315

[B30] Sigurdsson H. P. Yarnall A. J. Galna B. Lord S. Alcock L. Lawson R. A. et al. (2022). Gait-related metabolic covariance networks at rest in Parkinson’s disease. *Mov. Disord*. 37 1222–1234. 10.1002/mds.28977 35285068 PMC9314598

[B31] Sumarac S. Spencer K. A. Steiner L. A. Fearon C. Haniff E. A. Kühn A. A. et al. (2024). Interrogating basal ganglia circuit function in people with Parkinson’s disease and dystonia. *Elife* 12:R90454. 10.7554/eLife.90454 39190604 PMC11349293

[B32] Tinazzi M. Gandolfi M. Artusi C. A. Bannister K. Rukavina K. Brefel-Courbon C. et al. (2025). Advances in diagnosis, classification, and management of pain in Parkinson’s disease. *Lancet Neurol*. 24 331–347. 10.1016/S1474-4422(25)00033-X 40120617

[B33] Togo H. Nakamura T. Wakasugi N. Takahashi Y. Hanakawa T. (2023). Interactions across emotional, cognitive and subcortical motor networks underlying freezing of gait. *Neuroimage. Clin*. 37:103342. 10.1016/j.nicl.2023.103342 36739790 PMC9932566

[B34] Tzourio-Mazoyer N. Landeau B. Papathanassiou D. Crivello F. Etard O. Delcroix N. et al. (2002). Automated anatomical labeling of activations in SPM using a macroscopic anatomical parcellation of the MNI MRI single-subject brain. *Neuroimage* 15 273–289. 10.1006/nimg.2001.0978 11771995

[B35] Wang J. Shen Y. Peng J. Wang A. Wu X. Chen X. et al. (2021). Different functional connectivity modes of the right fronto-insular cortex in akinetic-rigid and tremor-dominant Parkinson’s disease. *Neurol. Sci*. 42 2937–2946. 10.1007/s10072-020-04917-1 33236247

[B36] Wu Y. Xu X. J. Sun X. Zhai H. Wang T. Cao X. B. et al. (2023). Integrated PET/MRI with 11C-CFT and 18F-FDG for levodopa response difference in Parkinson’s disease. *Behav. Brain Res*. 454:114609. 10.1016/j.bbr.2023.114609 37532003

[B37] Xu Z. Anderson K. N. Pavese N. (2022). Longitudinal studies of sleep disturbances in Parkinson’s disease. *Curr. Neurol. Neurosci. Rep*. 22 635–655. 10.1007/s11910-022-01223-5 36018498 PMC9617954

[B38] Yeo B. T. Krienen F. M. Sepulcre J. Sabuncu M. R. Lashkari D. Hollinshead M. et al. (2011). The organization of the human cerebral cortex estimated by intrinsic functional connectivity. *J. Neurophysiol*. 106 1125–1165. 10.1152/jn.00338.2011 21653723 PMC3174820

[B39] Zhang T. Yang R. Pan J. Huang S. (2023). Parkinson’s disease related depression and anxiety: A 22-year bibliometric analysis (2000-2022). *Neuropsychiatr. Dis. Treat*. 19 1477–1489. 10.2147/NDT.S403002 37404573 PMC10317541

[B40] Zimmerman M. Martinez J. H. Young D. Chelminski I. Dalrymple K. (2013). Severity classification on the hamilton depression rating scale. *J. Affect. Disord*. 150 384–388. 10.1016/j.jad.2013.04.028 23759278

