# Supplementary material for: Brain metabolic and network profiles characterized by comorbid depressive symptoms and sleep disturbances in Parkinson’s disease: an 18F-FDG PET study
Source: Front Aging Neurosci. 2026 Sep 9;18:1901625. doi: 10.3389/fnagi.2026.1901625 (PMC13597751; doi:10.3389/fnagi.2026.1901625)
Supplement: Supplementary file 1 [file Data_Sheet_1.docx]

 **Figure S1. Partial correlation scatter plots between regional brain metabolic values and HAMD scores in the PD-Dep group.**

**Figure S2. Partial correlation scatter plots between regional brain metabolic values and UPDRS-III scores in the PD-Dep group.**

| **TableS1 PD-Dep Group: Partial Correlation (UPDRS-III & HAMD vs. Brain Regions) with 95% CI** | | | | | |
| --- | --- | --- | --- | --- | --- |
| **Variable** | **Brain Region** | **r** | **95% CI** | **p value** | **df** |
| UPDRS-III | HAMD | 0.225 | [-0.401, 0.708] | 0.482 | 10 |
| **UPDRS-III** | **Precentral_L** | **-0.631** | **[-0.885, -0.090]** | **0.028** | **10** |
| **UPDRS-III** | **Precentral_R** | **-0.632** | **[-0.885, -0.091]** | **0.028** | **10** |
| **UPDRS-III** | **Frontal_Sup_L** | **-0.657** | **[-0.894, -0.133]** | **0.020** | **10** |
| **UPDRS-III** | **Frontal_Sup_R** | **-0.629** | **[-0.884, -0.086]** | **0.028** | **10** |
| UPDRS-III | Frontal_Sup_Orb_L | 0.073 | [-0.523, 0.621] | 0.822 | 10 |
| UPDRS-III | Frontal_Sup_Orb_R | 0.214 | [-0.410, 0.702] | 0.505 | 10 |
| **UPDRS-III** | **Frontal_Mid_L** | **-0.711** | **[-0.913, -0.232]** | **0.009** | **10** |
| **UPDRS-III** | **Frontal_Mid_R** | **-0.698** | **[-0.908, -0.207]** | **0.012** | **10** |
| UPDRS-III | Frontal_Mid_Orb_L | -0.088 | [-0.630, 0.512] | 0.786 | 10 |
| UPDRS-III | Frontal_Mid_Orb_R | 0.296 | [-0.335, 0.744] | 0.351 | 10 |
| UPDRS-III | Frontal_Inf_Oper_L | -0.267 | [-0.729, 0.362] | 0.402 | 10 |
| UPDRS-III | Frontal_Inf_Oper_R | -0.049 | [-0.606, 0.540] | 0.879 | 10 |
| UPDRS-III | Frontal_Inf_Tri_L | -0.445 | [-0.812, 0.173] | 0.147 | 10 |
| UPDRS-III | Frontal_Inf_Tri_R | -0.309 | [-0.750, 0.322] | 0.329 | 10 |
| UPDRS-III | Frontal_Inf_Orb_L | 0.095 | [-0.507, 0.634] | 0.768 | 10 |
| UPDRS-III | Frontal_Inf_Orb_R | 0.040 | [-0.546, 0.600] | 0.901 | 10 |
| UPDRS-III | Rolandic_Oper_L | 0.287 | [-0.343, 0.739] | 0.365 | 10 |
| UPDRS-III | Rolandic_Oper_R | 0.203 | [-0.420, 0.696] | 0.528 | 10 |
| UPDRS-III | Supp_Motor_Area_L | -0.519 | [-0.842, 0.078] | 0.084 | 10 |
| UPDRS-III | Supp_Motor_Area_R | -0.516 | [-0.841, 0.082] | 0.086 | 10 |
| UPDRS-III | Olfactory_L | -0.075 | [-0.622, 0.521] | 0.816 | 10 |
| UPDRS-III | Olfactory_R | 0.039 | [-0.547, 0.599] | 0.905 | 10 |
| UPDRS-III | Frontal_Sup_Medial_L | -0.548 | [-0.853, 0.038] | 0.065 | 10 |
| UPDRS-III | Frontal_Sup_Medial_R | -0.540 | [-0.850, 0.049] | 0.070 | 10 |
| UPDRS-III | Frontal_Med_Orb_L | 0.217 | [-0.408, 0.703] | 0.499 | 10 |
| UPDRS-III | Frontal_Med_Orb_R | 0.287 | [-0.343, 0.739] | 0.366 | 10 |
| UPDRS-III | Rectus_L | -0.043 | [-0.602, 0.544] | 0.894 | 10 |
| UPDRS-III | Rectus_R | -0.043 | [-0.602, 0.544] | 0.895 | 10 |
| UPDRS-III | Insula_L | 0.443 | [-0.176, 0.811] | 0.149 | 10 |
| UPDRS-III | Insula_R | 0.109 | [-0.496, 0.643] | 0.736 | 10 |
| UPDRS-III | Cingulum_Ant_L | 0.435 | [-0.185, 0.807] | 0.158 | 10 |
| UPDRS-III | Cingulum_Ant_R | 0.410 | [-0.214, 0.796] | 0.186 | 10 |
| UPDRS-III | Cingulum_Mid_L | 0.212 | [-0.412, 0.701] | 0.508 | 10 |
| UPDRS-III | Cingulum_Mid_R | 0.039 | [-0.547, 0.599] | 0.905 | 10 |
| UPDRS-III | Cingulum_Post_L | 0.158 | [-0.457, 0.671] | 0.625 | 10 |
| UPDRS-III | Cingulum_Post_R | 0.155 | [-0.460, 0.669] | 0.631 | 10 |
| UPDRS-III | Hippocampus_L | 0.459 | [-0.156, 0.818] | 0.134 | 10 |
| UPDRS-III | Hippocampus_R | 0.485 | [-0.123, 0.828] | 0.110 | 10 |
| UPDRS-III | ParaHippocampal_L | 0.321 | [-0.310, 0.756] | 0.309 | 10 |
| UPDRS-III | ParaHippocampal_R | 0.365 | [-0.264, 0.776] | 0.243 | 10 |
| UPDRS-III | Amygdala_L | 0.440 | [-0.179, 0.809] | 0.152 | 10 |
| UPDRS-III | Amygdala_R | 0.549 | [-0.036, 0.854] | 0.065 | 10 |
| UPDRS-III | Calcarine_L | 0.314 | [-0.317, 0.752] | 0.320 | 10 |
| UPDRS-III | Calcarine_R | 0.403 | [-0.222, 0.793] | 0.194 | 10 |
| UPDRS-III | Cuneus_L | 0.205 | [-0.418, 0.697] | 0.522 | 10 |
| UPDRS-III | Cuneus_R | 0.169 | [-0.448, 0.677] | 0.599 | 10 |
| UPDRS-III | Lingual_L | -0.094 | [-0.634, 0.507] | 0.772 | 10 |
| UPDRS-III | Lingual_R | 0.229 | [-0.397, 0.710] | 0.473 | 10 |
| UPDRS-III | Occipital_Sup_L | 0.149 | [-0.465, 0.666] | 0.643 | 10 |
| UPDRS-III | Occipital_Sup_R | 0.171 | [-0.447, 0.678] | 0.595 | 10 |
| UPDRS-III | Occipital_Mid_L | 0.256 | [-0.373, 0.724] | 0.421 | 10 |
| UPDRS-III | Occipital_Mid_R | 0.334 | [-0.297, 0.762] | 0.288 | 10 |
| UPDRS-III | Occipital_Inf_L | 0.110 | [-0.495, 0.643] | 0.734 | 10 |
| UPDRS-III | Occipital_Inf_R | 0.118 | [-0.489, 0.648] | 0.714 | 10 |
| UPDRS-III | Fusiform_L | -0.001 | [-0.575, 0.573] | 0.998 | 10 |
| UPDRS-III | Fusiform_R | 0.114 | [-0.492, 0.646] | 0.725 | 10 |
| **UPDRS-III** | **Postcentral_L** | **-0.668** | **[-0.898, -0.153]** | **0.018** | **10** |
| **UPDRS-III** | **Postcentral_R** | **-0.588** | **[-0.869, -0.021]** | **0.044** | **10** |
| UPDRS-III | Parietal_Sup_L | -0.443 | [-0.811, 0.176] | 0.149 | 10 |
| UPDRS-III | Parietal_Sup_R | -0.329 | [-0.759, 0.302] | 0.297 | 10 |
| **UPDRS-III** | **Parietal_Inf_L** | **-0.642** | **[-0.889, -0.108]** | **0.024** | **10** |
| **UPDRS-III** | **Parietal_Inf_R** | **-0.670** | **[-0.898, -0.156]** | **0.017** | **10** |
| **UPDRS-III** | **SupraMarginal_L** | **-0.606** | **[-0.875, -0.049]** | **0.037** | **10** |
| UPDRS-III | SupraMarginal_R | -0.285 | [-0.738, 0.345] | 0.369 | 10 |
| **UPDRS-III** | **Angular_L** | **-0.578** | **[-0.865, -0.006]** | **0.049** | **10** |
| UPDRS-III | Angular_R | -0.428 | [-0.804, 0.193] | 0.165 | 10 |
| UPDRS-III | Precuneus_L | -0.174 | [-0.680, 0.444] | 0.589 | 10 |
| UPDRS-III | Precuneus_R | -0.134 | [-0.657, 0.477] | 0.679 | 10 |
| UPDRS-III | Paracentral_Lobule_L | -0.442 | [-0.810, 0.177] | 0.150 | 10 |
| UPDRS-III | Paracentral_Lobule_R | -0.378 | [-0.782, 0.250] | 0.225 | 10 |
| UPDRS-III | Caudate_L | 0.080 | [-0.518, 0.625] | 0.804 | 10 |
| UPDRS-III | Caudate_R | 0.215 | [-0.409, 0.702] | 0.502 | 10 |
| UPDRS-III | Putamen_L | -0.270 | [-0.731, 0.360] | 0.396 | 10 |
| UPDRS-III | Putamen_R | -0.281 | [-0.736, 0.349] | 0.376 | 10 |
| UPDRS-III | Pallidum_L | -0.303 | [-0.747, 0.328] | 0.339 | 10 |
| UPDRS-III | Pallidum_R | 0.061 | [-0.532, 0.613] | 0.851 | 10 |
| UPDRS-III | Thalamus_L | -0.080 | [-0.625, 0.518] | 0.805 | 10 |
| UPDRS-III | Thalamus_R | 0.007 | [-0.569, 0.579] | 0.983 | 10 |
| UPDRS-III | Heschl_L | 0.304 | [-0.327, 0.747] | 0.336 | 10 |
| UPDRS-III | Heschl_R | 0.015 | [-0.564, 0.584] | 0.964 | 10 |
| UPDRS-III | Temporal_Sup_L | 0.103 | [-0.500, 0.639] | 0.750 | 10 |
| UPDRS-III | Temporal_Sup_R | 0.285 | [-0.345, 0.738] | 0.369 | 10 |
| UPDRS-III | Temporal_Pole_Sup_L | 0.330 | [-0.301, 0.760] | 0.296 | 10 |
| UPDRS-III | Temporal_Pole_Sup_R | 0.443 | [-0.176, 0.811] | 0.149 | 10 |
| UPDRS-III | Temporal_Mid_L | 0.146 | [-0.467, 0.664] | 0.652 | 10 |
| UPDRS-III | Temporal_Mid_R | 0.264 | [-0.365, 0.728] | 0.407 | 10 |
| UPDRS-III | Temporal_Pole_Mid_L | 0.160 | [-0.456, 0.672] | 0.620 | 10 |
| UPDRS-III | Temporal_Pole_Mid_R | 0.400 | [-0.226, 0.792] | 0.198 | 10 |
| UPDRS-III | Temporal_Inf_L | 0.066 | [-0.528, 0.617] | 0.838 | 10 |
| UPDRS-III | Temporal_Inf_R | 0.173 | [-0.445, 0.679] | 0.591 | 10 |
| HAMD | UPDRS-III | 0.225 | [-0.401, 0.708] | 0.482 | 10 |
| HAMD | Precentral_L | -0.243 | [-0.717, 0.385] | 0.446 | 10 |
| HAMD | Precentral_R | -0.316 | [-0.753, 0.315] | 0.318 | 10 |
| HAMD | Frontal_Sup_L | -0.129 | [-0.654, 0.480] | 0.689 | 10 |
| HAMD | Frontal_Sup_R | -0.241 | [-0.716, 0.386] | 0.451 | 10 |
| HAMD | Frontal_Sup_Orb_L | 0.040 | [-0.546, 0.600] | 0.902 | 10 |
| HAMD | Frontal_Sup_Orb_R | -0.196 | [-0.692, 0.426] | 0.541 | 10 |
| HAMD | Frontal_Mid_L | -0.194 | [-0.691, 0.427] | 0.545 | 10 |
| HAMD | Frontal_Mid_R | -0.169 | [-0.677, 0.448] | 0.598 | 10 |
| HAMD | Frontal_Mid_Orb_L | -0.204 | [-0.696, 0.419] | 0.525 | 10 |
| HAMD | Frontal_Mid_Orb_R | -0.122 | [-0.650, 0.486] | 0.705 | 10 |
| HAMD | Frontal_Inf_Oper_L | -0.421 | [-0.801, 0.202] | 0.173 | 10 |
| HAMD | Frontal_Inf_Oper_R | -0.415 | [-0.799, 0.209] | 0.180 | 10 |
| HAMD | Frontal_Inf_Tri_L | -0.349 | [-0.769, 0.281] | 0.266 | 10 |
| HAMD | Frontal_Inf_Tri_R | -0.484 | [-0.828, 0.124] | 0.111 | 10 |
| HAMD | Frontal_Inf_Orb_L | -0.330 | [-0.760, 0.301] | 0.295 | 10 |
| HAMD | Frontal_Inf_Orb_R | -0.362 | [-0.775, 0.267] | 0.247 | 10 |
| HAMD | Rolandic_Oper_L | -0.239 | [-0.715, 0.388] | 0.454 | 10 |
| HAMD | Rolandic_Oper_R | -0.325 | [-0.758, 0.306] | 0.302 | 10 |
| HAMD | Supp_Motor_Area_L | -0.222 | [-0.706, 0.403] | 0.488 | 10 |
| HAMD | Supp_Motor_Area_R | -0.214 | [-0.702, 0.410] | 0.505 | 10 |
| HAMD | Olfactory_L | -0.384 | [-0.785, 0.244] | 0.218 | 10 |
| HAMD | Olfactory_R | -0.516 | [-0.841, 0.082] | 0.086 | 10 |
| HAMD | Frontal_Sup_Medial_L | -0.061 | [-0.613, 0.532] | 0.851 | 10 |
| HAMD | Frontal_Sup_Medial_R | -0.197 | [-0.693, 0.425] | 0.539 | 10 |
| HAMD | Frontal_Med_Orb_L | -0.196 | [-0.692, 0.426] | 0.542 | 10 |
| HAMD | Frontal_Med_Orb_R | -0.348 | [-0.768, 0.282] | 0.268 | 10 |
| HAMD | Rectus_L | -0.296 | [-0.744, 0.335] | 0.350 | 10 |
| HAMD | Rectus_R | -0.430 | [-0.805, 0.191] | 0.163 | 10 |
| HAMD | Insula_L | -0.268 | [-0.730, 0.362] | 0.401 | 10 |
| HAMD | Insula_R | -0.523 | [-0.844, 0.073] | 0.081 | 10 |
| HAMD | Cingulum_Ant_L | -0.233 | [-0.712, 0.394] | 0.465 | 10 |
| HAMD | Cingulum_Ant_R | -0.301 | [-0.746, 0.330] | 0.341 | 10 |
| HAMD | Cingulum_Mid_L | -0.040 | [-0.600, 0.546] | 0.902 | 10 |
| HAMD | Cingulum_Mid_R | -0.084 | [-0.628, 0.515] | 0.795 | 10 |
| HAMD | Cingulum_Post_L | 0.018 | [-0.562, 0.586] | 0.955 | 10 |
| HAMD | Cingulum_Post_R | 0.050 | [-0.539, 0.606] | 0.878 | 10 |
| HAMD | Hippocampus_L | -0.295 | [-0.743, 0.336] | 0.352 | 10 |
| HAMD | Hippocampus_R | -0.303 | [-0.747, 0.328] | 0.338 | 10 |
| HAMD | ParaHippocampal_L | -0.229 | [-0.710, 0.397] | 0.474 | 10 |
| HAMD | ParaHippocampal_R | -0.223 | [-0.706, 0.402] | 0.486 | 10 |
| HAMD | Amygdala_L | -0.264 | [-0.728, 0.365] | 0.406 | 10 |
| HAMD | Amygdala_R | -0.210 | [-0.700, 0.414] | 0.512 | 10 |
| HAMD | Calcarine_L | -0.215 | [-0.702, 0.409] | 0.501 | 10 |
| HAMD | Calcarine_R | -0.138 | [-0.660, 0.473] | 0.669 | 10 |
| HAMD | Cuneus_L | -0.450 | [-0.814, 0.167] | 0.142 | 10 |
| HAMD | Cuneus_R | -0.534 | [-0.848, 0.058] | 0.074 | 10 |
| HAMD | Lingual_L | -0.449 | [-0.813, 0.168] | 0.143 | 10 |
| HAMD | Lingual_R | -0.088 | [-0.630, 0.512] | 0.785 | 10 |
| HAMD | Occipital_Sup_L | -0.428 | [-0.804, 0.193] | 0.165 | 10 |
| HAMD | Occipital_Sup_R | -0.493 | [-0.832, 0.113] | 0.103 | 10 |
| HAMD | Occipital_Mid_L | -0.235 | [-0.713, 0.392] | 0.461 | 10 |
| HAMD | Occipital_Mid_R | -0.241 | [-0.716, 0.386] | 0.451 | 10 |
| HAMD | Occipital_Inf_L | 0.252 | [-0.376, 0.722] | 0.429 | 10 |
| HAMD | Occipital_Inf_R | 0.119 | [-0.488, 0.649] | 0.712 | 10 |
| HAMD | Fusiform_L | -0.258 | [-0.725, 0.371] | 0.419 | 10 |
| HAMD | Fusiform_R | -0.229 | [-0.710, 0.397] | 0.473 | 10 |
| HAMD | Postcentral_L | -0.418 | [-0.800, 0.205] | 0.177 | 10 |
| **HAMD** | **Postcentral_R** | **-0.586** | **[-0.868, -0.018]** | **0.045** | **10** |
| HAMD | Parietal_Sup_L | -0.347 | [-0.768, 0.283] | 0.269 | 10 |
| HAMD | Parietal_Sup_R | -0.443 | [-0.811, 0.176] | 0.150 | 10 |
| HAMD | Parietal_Inf_L | -0.410 | [-0.796, 0.214] | 0.186 | 10 |
| HAMD | Parietal_Inf_R | -0.377 | [-0.782, 0.251] | 0.227 | 10 |
| **HAMD** | **SupraMarginal_L** | **-0.698** | **[-0.908, -0.207]** | **0.012** | **10** |
| HAMD | SupraMarginal_R | -0.493 | [-0.832, 0.113] | 0.104 | 10 |
| HAMD | Angular_L | -0.415 | [-0.799, 0.209] | 0.179 | 10 |
| HAMD | Angular_R | -0.523 | [-0.844, 0.073] | 0.081 | 10 |
| HAMD | Precuneus_L | -0.365 | [-0.776, 0.264] | 0.243 | 10 |
| HAMD | Precuneus_R | -0.397 | [-0.791, 0.229] | 0.201 | 10 |
| HAMD | Paracentral_Lobule_L | -0.285 | [-0.738, 0.345] | 0.369 | 10 |
| HAMD | Paracentral_Lobule_R | -0.225 | [-0.708, 0.401] | 0.483 | 10 |
| HAMD | Caudate_L | -0.487 | [-0.829, 0.121] | 0.108 | 10 |
| HAMD | Caudate_R | -0.528 | [-0.846, 0.066] | 0.078 | 10 |
| **HAMD** | **Putamen_L** | **-0.671** | **[-0.899, -0.158]** | **0.017** | **10** |
| **HAMD** | **Putamen_R** | **-0.828** | **[-0.950, -0.484]** | **0.001** | **10** |
| HAMD | Pallidum_L | -0.411 | [-0.797, 0.213] | 0.185 | 10 |
| **HAMD** | **Pallidum_R** | **-0.589** | **[-0.869, -0.023]** | **0.044** | **10** |
| **HAMD** | **Thalamus_L** | **-0.649** | **[-0.891, -0.120]** | **0.022** | **10** |
| **HAMD** | **Thalamus_R** | **-0.640** | **[-0.888, -0.104]** | **0.025** | **10** |
| HAMD | Heschl_L | -0.339 | [-0.764, 0.292] | 0.281 | 10 |
| HAMD | Heschl_R | -0.445 | [-0.812, 0.173] | 0.147 | 10 |
| HAMD | Temporal_Sup_L | -0.496 | [-0.833, 0.109] | 0.101 | 10 |
| HAMD | Temporal_Sup_R | -0.408 | [-0.796, 0.217] | 0.188 | 10 |
| HAMD | Temporal_Pole_Sup_L | -0.468 | [-0.821, 0.145] | 0.125 | 10 |
| HAMD | Temporal_Pole_Sup_R | -0.345 | [-0.767, 0.285] | 0.273 | 10 |
| HAMD | Temporal_Mid_L | -0.417 | [-0.800, 0.206] | 0.177 | 10 |
| HAMD | Temporal_Mid_R | -0.251 | [-0.721, 0.377] | 0.432 | 10 |
| HAMD | Temporal_Pole_Mid_L | -0.480 | [-0.826, 0.130] | 0.115 | 10 |
| HAMD | Temporal_Pole_Mid_R | -0.472 | [-0.823, 0.140] | 0.121 | 10 |
| HAMD | Temporal_Inf_L | -0.273 | [-0.732, 0.357] | 0.390 | 10 |
| HAMD | Temporal_Inf_R | -0.159 | [-0.672, 0.457] | 0.621 | 10 |
|  |  |  |  |  |  |
| *Note: N = 16, df = 10, number of controlled variables k = 4. 95% CI calculated via Fisher z transformation (SE = 1/sqrt(df-1)). r = partial correlation coefficient. Highlighted rows indicate p < 0.05.* | | | | | |

-

**Figure S3. Partial correlation scatter plots between regional brain metabolic values and PSQI scores in the PD-SD group. Scatter plots illustrating positive and negative correlations, respectively.**
-

**Figure S4. Partial correlation scatter plots between regional brain metabolic values and UPDRS-III scores in the PD-SD group. Scatter plots illustrating positive and negative correlations, respectively.**

| **TableS2 PD-SD Group: Partial Correlation (UPDRS-III & PSQI vs. Brain Regions) with 95% CI** | | | | | |
| --- | --- | --- | --- | --- | --- |
| **Variable** | **Brain Region** | **r** | **95% CI** | **p value** | **df** |
| UPDRS-III | PSQI | 0.043 | [-0.447, 0.513] | 0.871 | 15 |
| UPDRS-III | Precentral_L | -0.389 | [-0.733, 0.113] | 0.123 | 15 |
| UPDRS-III | Precentral_R | -0.361 | [-0.717, 0.145] | 0.155 | 15 |
| UPDRS-III | Frontal_Sup_L | -0.399 | [-0.738, 0.101] | 0.112 | 15 |
| UPDRS-III | Frontal_Sup_R | -0.315 | [-0.691, 0.195] | 0.218 | 15 |
| UPDRS-III | Frontal_Sup_Orb_L | 0.213 | [-0.298, 0.629] | 0.412 | 15 |
| UPDRS-III | Frontal_Sup_Orb_R | 0.418 | [-0.078, 0.748] | 0.095 | 15 |
| **UPDRS-III** | **Frontal_Mid_L** | **-0.529** | **[-0.805, -0.065]** | **0.029** | **15** |
| **UPDRS-III** | **Frontal_Mid_R** | **-0.500** | **[-0.791, -0.025]** | **0.041** | **15** |
| UPDRS-III | Frontal_Mid_Orb_L | 0.073 | [-0.422, 0.535] | 0.782 | 15 |
| UPDRS-III | Frontal_Mid_Orb_R | 0.460 | [-0.027, 0.770] | 0.064 | 15 |
| UPDRS-III | Frontal_Inf_Oper_L | -0.127 | [-0.573, 0.377] | 0.628 | 15 |
| UPDRS-III | Frontal_Inf_Oper_R | 0.085 | [-0.412, 0.543] | 0.746 | 15 |
| UPDRS-III | Frontal_Inf_Tri_L | -0.358 | [-0.716, 0.148] | 0.159 | 15 |
| UPDRS-III | Frontal_Inf_Tri_R | -0.161 | [-0.596, 0.346] | 0.537 | 15 |
| UPDRS-III | Frontal_Inf_Orb_L | 0.216 | [-0.295, 0.631] | 0.406 | 15 |
| UPDRS-III | Frontal_Inf_Orb_R | 0.143 | [-0.363, 0.584] | 0.583 | 15 |
| UPDRS-III | Rolandic_Oper_L | 0.474 | [-0.009, 0.778] | 0.054 | 15 |
| UPDRS-III | Rolandic_Oper_R | 0.390 | [-0.112, 0.733] | 0.122 | 15 |
| UPDRS-III | Supp_Motor_Area_L | -0.297 | [-0.681, 0.214] | 0.247 | 15 |
| UPDRS-III | Supp_Motor_Area_R | -0.285 | [-0.673, 0.227] | 0.267 | 15 |
| UPDRS-III | Olfactory_L | 0.219 | [-0.292, 0.633] | 0.399 | 15 |
| UPDRS-III | Olfactory_R | 0.321 | [-0.189, 0.694] | 0.208 | 15 |
| UPDRS-III | Frontal_Sup_Medial_L | -0.434 | [-0.757, 0.059] | 0.082 | 15 |
| UPDRS-III | Frontal_Sup_Medial_R | -0.423 | [-0.751, 0.072] | 0.090 | 15 |
| UPDRS-III | Frontal_Med_Orb_L | 0.360 | [-0.146, 0.717] | 0.156 | 15 |
| UPDRS-III | Frontal_Med_Orb_R | 0.429 | [-0.065, 0.754] | 0.086 | 15 |
| UPDRS-III | Rectus_L | 0.146 | [-0.360, 0.586] | 0.575 | 15 |
| UPDRS-III | Rectus_R | 0.179 | [-0.330, 0.607] | 0.491 | 15 |
| **UPDRS-III** | **Insula_L** | **0.549** | **[0.093, 0.815]** | **0.023** | **15** |
| UPDRS-III | Insula_R | 0.315 | [-0.195, 0.691] | 0.219 | 15 |
| **UPDRS-III** | **Cingulum_Ant_L** | **0.525** | **[0.059, 0.803]** | **0.030** | **15** |
| UPDRS-III | Cingulum_Ant_R | 0.460 | [-0.027, 0.770] | 0.063 | 15 |
| UPDRS-III | Cingulum_Mid_L | 0.342 | [-0.166, 0.707] | 0.179 | 15 |
| UPDRS-III | Cingulum_Mid_R | 0.173 | [-0.336, 0.603] | 0.507 | 15 |
| UPDRS-III | Cingulum_Post_L | 0.155 | [-0.352, 0.592] | 0.552 | 15 |
| UPDRS-III | Cingulum_Post_R | 0.144 | [-0.362, 0.584] | 0.583 | 15 |
| **UPDRS-III** | **Hippocampus_L** | **0.596** | **[0.162, 0.837]** | **0.012** | **15** |
| **UPDRS-III** | **Hippocampus_R** | **0.675** | **[0.288, 0.873]** | **0.003** | **15** |
| UPDRS-III | ParaHippocampal_L | 0.464 | [-0.021, 0.772] | 0.060 | 15 |
| **UPDRS-III** | **ParaHippocampal_R** | **0.561** | **[0.110, 0.820]** | **0.019** | **15** |
| **UPDRS-III** | **Amygdala_L** | **0.511** | **[0.040, 0.796]** | **0.036** | **15** |
| **UPDRS-III** | **Amygdala_R** | **0.613** | **[0.188, 0.845]** | **0.009** | **15** |
| **UPDRS-III** | **Calcarine_L** | **0.557** | **[0.104, 0.819]** | **0.020** | **15** |
| UPDRS-III | Calcarine_R | 0.471 | [-0.012, 0.776] | 0.057 | 15 |
| UPDRS-III | Cuneus_L | 0.433 | [-0.060, 0.756] | 0.082 | 15 |
| UPDRS-III | Cuneus_R | 0.439 | [-0.053, 0.759] | 0.078 | 15 |
| UPDRS-III | Lingual_L | 0.263 | [-0.249, 0.660] | 0.308 | 15 |
| UPDRS-III | Lingual_R | 0.379 | [-0.124, 0.727] | 0.133 | 15 |
| UPDRS-III | Occipital_Sup_L | 0.358 | [-0.148, 0.716] | 0.158 | 15 |
| UPDRS-III | Occipital_Sup_R | 0.419 | [-0.077, 0.749] | 0.094 | 15 |
| UPDRS-III | Occipital_Mid_L | 0.462 | [-0.024, 0.771] | 0.062 | 15 |
| UPDRS-III | Occipital_Mid_R | 0.473 | [-0.010, 0.777] | 0.055 | 15 |
| UPDRS-III | Occipital_Inf_L | 0.274 | [-0.238, 0.667] | 0.288 | 15 |
| UPDRS-III | Occipital_Inf_R | 0.241 | [-0.271, 0.647] | 0.352 | 15 |
| UPDRS-III | Fusiform_L | 0.248 | [-0.264, 0.651] | 0.337 | 15 |
| UPDRS-III | Fusiform_R | 0.344 | [-0.164, 0.708] | 0.177 | 15 |
| UPDRS-III | Postcentral_L | -0.361 | [-0.717, 0.145] | 0.155 | 15 |
| UPDRS-III | Postcentral_R | -0.250 | [-0.652, 0.262] | 0.333 | 15 |
| UPDRS-III | Parietal_Sup_L | -0.211 | [-0.628, 0.300] | 0.415 | 15 |
| UPDRS-III | Parietal_Sup_R | -0.147 | [-0.586, 0.359] | 0.574 | 15 |
| UPDRS-III | Parietal_Inf_L | -0.321 | [-0.694, 0.189] | 0.208 | 15 |
| UPDRS-III | Parietal_Inf_R | -0.318 | [-0.693, 0.192] | 0.214 | 15 |
| UPDRS-III | SupraMarginal_L | -0.216 | [-0.631, 0.295] | 0.405 | 15 |
| UPDRS-III | SupraMarginal_R | 0.040 | [-0.449, 0.511] | 0.878 | 15 |
| UPDRS-III | Angular_L | -0.281 | [-0.671, 0.231] | 0.275 | 15 |
| UPDRS-III | Angular_R | -0.166 | [-0.599, 0.342] | 0.525 | 15 |
| UPDRS-III | Precuneus_L | 0.032 | [-0.456, 0.505] | 0.904 | 15 |
| UPDRS-III | Precuneus_R | 0.062 | [-0.432, 0.527] | 0.813 | 15 |
| UPDRS-III | Paracentral_Lobule_L | -0.214 | [-0.630, 0.297] | 0.411 | 15 |
| UPDRS-III | Paracentral_Lobule_R | -0.212 | [-0.629, 0.299] | 0.413 | 15 |
| UPDRS-III | Caudate_L | 0.437 | [-0.055, 0.758] | 0.080 | 15 |
| **UPDRS-III** | **Caudate_R** | **0.498** | **[0.023, 0.790]** | **0.042** | **15** |
| UPDRS-III | Putamen_L | 0.166 | [-0.342, 0.599] | 0.525 | 15 |
| UPDRS-III | Putamen_R | 0.186 | [-0.324, 0.612] | 0.475 | 15 |
| UPDRS-III | Pallidum_L | 0.097 | [-0.402, 0.552] | 0.711 | 15 |
| UPDRS-III | Pallidum_R | 0.350 | [-0.157, 0.711] | 0.168 | 15 |
| UPDRS-III | Thalamus_L | 0.214 | [-0.297, 0.630] | 0.409 | 15 |
| UPDRS-III | Thalamus_R | 0.303 | [-0.208, 0.684] | 0.238 | 15 |
| UPDRS-III | Heschl_L | 0.436 | [-0.056, 0.758] | 0.080 | 15 |
| UPDRS-III | Heschl_R | 0.243 | [-0.269, 0.648] | 0.348 | 15 |
| UPDRS-III | Temporal_Sup_L | 0.317 | [-0.193, 0.692] | 0.215 | 15 |
| UPDRS-III | Temporal_Sup_R | 0.448 | [-0.042, 0.764] | 0.071 | 15 |
| UPDRS-III | Temporal_Pole_Sup_L | 0.382 | [-0.121, 0.729] | 0.130 | 15 |
| UPDRS-III | Temporal_Pole_Sup_R | 0.439 | [-0.053, 0.759] | 0.078 | 15 |
| UPDRS-III | Temporal_Mid_L | 0.398 | [-0.102, 0.738] | 0.114 | 15 |
| UPDRS-III | Temporal_Mid_R | 0.463 | [-0.023, 0.772] | 0.061 | 15 |
| UPDRS-III | Temporal_Pole_Mid_L | 0.285 | [-0.227, 0.673] | 0.268 | 15 |
| **UPDRS-III** | **Temporal_Pole_Mid_R** | **0.551** | **[0.096, 0.816]** | **0.022** | **15** |
| UPDRS-III | Temporal_Inf_L | 0.228 | [-0.284, 0.639] | 0.380 | 15 |
| UPDRS-III | Temporal_Inf_R | 0.296 | [-0.215, 0.680] | 0.249 | 15 |
| PSQI | UPDRS-III | 0.043 | [-0.447, 0.513] | 0.871 | 15 |
| **PSQI** | **Precentral_L** | **-0.589** | **[-0.834, -0.151]** | **0.013** | **15** |
| **PSQI** | **Precentral_R** | **-0.500** | **[-0.791, -0.025]** | **0.041** | **15** |
| **PSQI** | **Frontal_Sup_L** | **-0.556** | **[-0.818, -0.103]** | **0.020** | **15** |
| **PSQI** | **Frontal_Sup_R** | **-0.550** | **[-0.815, -0.094]** | **0.022** | **15** |
| PSQI | Frontal_Sup_Orb_L | 0.170 | [-0.338, 0.601] | 0.514 | 15 |
| PSQI | Frontal_Sup_Orb_R | 0.207 | [-0.304, 0.625] | 0.425 | 15 |
| **PSQI** | **Frontal_Mid_L** | **-0.525** | **[-0.803, -0.059]** | **0.030** | **15** |
| **PSQI** | **Frontal_Mid_R** | **-0.531** | **[-0.806, -0.068]** | **0.028** | **15** |
| PSQI | Frontal_Mid_Orb_L | 0.303 | [-0.208, 0.684] | 0.237 | 15 |
| PSQI | Frontal_Mid_Orb_R | 0.299 | [-0.212, 0.682] | 0.244 | 15 |
| PSQI | Frontal_Inf_Oper_L | -0.077 | [-0.538, 0.419] | 0.770 | 15 |
| PSQI | Frontal_Inf_Oper_R | 0.319 | [-0.191, 0.693] | 0.212 | 15 |
| PSQI | Frontal_Inf_Tri_L | -0.261 | [-0.659, 0.251] | 0.312 | 15 |
| PSQI | Frontal_Inf_Tri_R | 0.356 | [-0.150, 0.714] | 0.160 | 15 |
| **PSQI** | **Frontal_Inf_Orb_L** | **0.520** | **[0.052, 0.801]** | **0.032** | **15** |
| PSQI | Frontal_Inf_Orb_R | 0.413 | [-0.084, 0.746] | 0.099 | 15 |
| PSQI | Rolandic_Oper_L | 0.470 | [-0.014, 0.775] | 0.057 | 15 |
| PSQI | Rolandic_Oper_R | 0.455 | [-0.033, 0.768] | 0.067 | 15 |
| **PSQI** | **Supp_Motor_Area_L** | **-0.547** | **[-0.814, -0.090]** | **0.023** | **15** |
| **PSQI** | **Supp_Motor_Area_R** | **-0.541** | **[-0.811, -0.082]** | **0.025** | **15** |
| PSQI | Olfactory_L | 0.203 | [-0.308, 0.623] | 0.434 | 15 |
| PSQI | Olfactory_R | 0.171 | [-0.337, 0.602] | 0.511 | 15 |
| **PSQI** | **Frontal_Sup_Medial_L** | **-0.570** | **[-0.825, -0.123]** | **0.017** | **15** |
| **PSQI** | **Frontal_Sup_Medial_R** | **-0.498** | **[-0.790, -0.023]** | **0.042** | **15** |
| PSQI | Frontal_Med_Orb_L | 0.347 | [-0.160, 0.709] | 0.173 | 15 |
| PSQI | Frontal_Med_Orb_R | 0.342 | [-0.166, 0.707] | 0.179 | 15 |
| PSQI | Rectus_L | 0.234 | [-0.278, 0.642] | 0.365 | 15 |
| PSQI | Rectus_R | 0.192 | [-0.318, 0.616] | 0.460 | 15 |
| PSQI | Insula_L | 0.434 | [-0.059, 0.757] | 0.082 | 15 |
| PSQI | Insula_R | 0.244 | [-0.268, 0.649] | 0.346 | 15 |
| PSQI | Cingulum_Ant_L | 0.439 | [-0.053, 0.759] | 0.078 | 15 |
| PSQI | Cingulum_Ant_R | 0.452 | [-0.037, 0.766] | 0.069 | 15 |
| PSQI | Cingulum_Mid_L | 0.251 | [-0.261, 0.653] | 0.331 | 15 |
| PSQI | Cingulum_Mid_R | 0.161 | [-0.346, 0.596] | 0.537 | 15 |
| PSQI | Cingulum_Post_L | 0.361 | [-0.145, 0.717] | 0.155 | 15 |
| PSQI | Cingulum_Post_R | 0.374 | [-0.130, 0.724] | 0.139 | 15 |
| PSQI | Hippocampus_L | 0.406 | [-0.093, 0.742] | 0.106 | 15 |
| PSQI | Hippocampus_R | 0.190 | [-0.320, 0.615] | 0.466 | 15 |
| PSQI | ParaHippocampal_L | 0.448 | [-0.042, 0.764] | 0.071 | 15 |
| PSQI | ParaHippocampal_R | 0.264 | [-0.248, 0.661] | 0.305 | 15 |
| **PSQI** | **Amygdala_L** | **0.661** | **[0.264, 0.866]** | **0.004** | **15** |
| **PSQI** | **Amygdala_R** | **0.532** | **[0.069, 0.806]** | **0.028** | **15** |
| PSQI | Calcarine_L | 0.104 | [-0.396, 0.557] | 0.692 | 15 |
| PSQI | Calcarine_R | 0.165 | [-0.343, 0.598] | 0.528 | 15 |
| PSQI | Cuneus_L | 0.296 | [-0.215, 0.680] | 0.248 | 15 |
| PSQI | Cuneus_R | 0.304 | [-0.207, 0.685] | 0.236 | 15 |
| PSQI | Lingual_L | -0.240 | [-0.646, 0.272] | 0.353 | 15 |
| PSQI | Lingual_R | 0.231 | [-0.281, 0.641] | 0.372 | 15 |
| PSQI | Occipital_Sup_L | 0.182 | [-0.327, 0.609] | 0.485 | 15 |
| PSQI | Occipital_Sup_R | 0.246 | [-0.266, 0.650] | 0.340 | 15 |
| PSQI | Occipital_Mid_L | 0.021 | [-0.464, 0.497] | 0.938 | 15 |
| PSQI | Occipital_Mid_R | 0.047 | [-0.444, 0.516] | 0.858 | 15 |
| PSQI | Occipital_Inf_L | -0.359 | [-0.716, 0.147] | 0.157 | 15 |
| PSQI | Occipital_Inf_R | -0.248 | [-0.651, 0.264] | 0.337 | 15 |
| PSQI | Fusiform_L | -0.168 | [-0.600, 0.340] | 0.519 | 15 |
| PSQI | Fusiform_R | -0.130 | [-0.575, 0.374] | 0.619 | 15 |
| PSQI | Postcentral_L | -0.475 | [-0.778, 0.007] | 0.054 | 15 |
| PSQI | Postcentral_R | -0.230 | [-0.640, 0.282] | 0.374 | 15 |
| PSQI | Parietal_Sup_L | -0.438 | [-0.759, 0.054] | 0.079 | 15 |
| PSQI | Parietal_Sup_R | -0.345 | [-0.708, 0.163] | 0.175 | 15 |
| PSQI | Parietal_Inf_L | -0.450 | [-0.765, 0.039] | 0.070 | 15 |
| PSQI | Parietal_Inf_R | -0.426 | [-0.753, 0.069] | 0.088 | 15 |
| PSQI | SupraMarginal_L | -0.200 | [-0.621, 0.310] | 0.442 | 15 |
| PSQI | SupraMarginal_R | 0.411 | [-0.087, 0.745] | 0.102 | 15 |
| PSQI | Angular_L | -0.311 | [-0.689, 0.199] | 0.224 | 15 |
| PSQI | Angular_R | -0.107 | [-0.559, 0.394] | 0.683 | 15 |
| PSQI | Precuneus_L | -0.227 | [-0.638, 0.285] | 0.380 | 15 |
| PSQI | Precuneus_R | -0.154 | [-0.591, 0.353] | 0.555 | 15 |
| PSQI | Paracentral_Lobule_L | -0.482 | [-0.782, -0.002] | 0.050 | 15 |
| PSQI | Paracentral_Lobule_R | -0.461 | [-0.771, 0.025] | 0.062 | 15 |
| PSQI | Caudate_L | 0.086 | [-0.412, 0.544] | 0.742 | 15 |
| PSQI | Caudate_R | 0.264 | [-0.248, 0.661] | 0.306 | 15 |
| PSQI | Putamen_L | -0.017 | [-0.494, 0.467] | 0.949 | 15 |
| PSQI | Putamen_R | -0.091 | [-0.548, 0.407] | 0.729 | 15 |
| PSQI | Pallidum_L | -0.149 | [-0.588, 0.357] | 0.569 | 15 |
| PSQI | Pallidum_R | 0.007 | [-0.475, 0.486] | 0.978 | 15 |
| PSQI | Thalamus_L | -0.079 | [-0.539, 0.417] | 0.764 | 15 |
| PSQI | Thalamus_R | 0.115 | [-0.387, 0.564] | 0.660 | 15 |
| **PSQI** | **Heschl_L** | **0.556** | **[0.103, 0.818]** | **0.021** | **15** |
| **PSQI** | **Heschl_R** | **0.488** | **[0.010, 0.785]** | **0.047** | **15** |
| **PSQI** | **Temporal_Sup_L** | **0.515** | **[0.046, 0.798]** | **0.034** | **15** |
| PSQI | Temporal_Sup_R | 0.474 | [-0.009, 0.778] | 0.055 | 15 |
| **PSQI** | **Temporal_Pole_Sup_L** | **0.735** | **[0.393, 0.898]** | **0.001** | **15** |
| **PSQI** | **Temporal_Pole_Sup_R** | **0.690** | **[0.313, 0.879]** | **0.002** | **15** |
| PSQI | Temporal_Mid_L | 0.334 | [-0.175, 0.702] | 0.190 | 15 |
| PSQI | Temporal_Mid_R | 0.178 | [-0.331, 0.607] | 0.493 | 15 |
| **PSQI** | **Temporal_Pole_Mid_L** | **0.542** | **[0.083, 0.811]** | **0.025** | **15** |
| **PSQI** | **Temporal_Pole_Mid_R** | **0.528** | **[0.063, 0.804]** | **0.029** | **15** |
| PSQI | Temporal_Inf_L | 0.307 | [-0.204, 0.686] | 0.231 | 15 |
| PSQI | Temporal_Inf_R | 0.160 | [-0.347, 0.595] | 0.539 | 15 |
|  |  |  |  |  |  |
| *Note: N = 21, df = 15, number of controlled variables k = 4. 95% CI calculated via Fisher z transformation (SE = 1/sqrt(df-1)). r = partial correlation coefficient. Highlighted rows indicate p < 0.05.* | | | | | |
|  |  |  |  |  |  |
